# Complement pathway activation mediates pancreatic cancer–induced muscle wasting and pathological remodeling

**DOI:** 10.1172/JCI178806

**Published:** 2025-04-08

**Authors:** Andrew C. D’Lugos, Jeremy B. Ducharme, Chandler S. Callaway, Jose G. Trevino, Carl Atkinson, Sarah M. Judge, Andrew R. Judge

**Affiliations:** 1 Department of Physical Therapy and; 2 Myology Institute, University of Florida, Gainesville, Florida, USA.; 3 University of Florida Health Cancer Center, Gainesville, Florida, USA.; 4 Department of Surgery, Virginia Commonwealth University School of Medicine, Richmond, Virginia, USA.; 5 Division of Pulmonary Medicine, University of Florida, Gainesville, Florida, USA.

**Keywords:** Muscle biology, Oncology, Cancer, Muscle, Proteomics

## Abstract

Cancer cachexia is a multifactorial condition characterized by skeletal muscle wasting that impairs quality of life and longevity for many cancer patients. A greater understanding of the molecular etiology of this condition is needed for effective therapies to be developed. We performed a quantitative proteomic analysis of skeletal muscle from cachectic pancreatic ductal adenocarcinoma (PDAC) patients and non-cancer controls, followed by immunohistochemical analyses of muscle cross sections. These data provide evidence of a local inflammatory response in muscles of cachectic PDAC patients, including an accumulation of plasma proteins and recruitment of immune cells into muscle that may promote the pathological remodeling of muscle. Our data further support the complement system as a potential mediator of these processes, which we tested by injecting murine pancreatic cancer cells into wild-type mice and mice with genetic deletion of the central complement component 3 (C3^–/–^ mice). Compared with wild-type mice, C3^–/–^ mice showed attenuated tumor-induced muscle wasting and dysfunction and reduced immune cell recruitment and fibrotic remodeling of muscle. These studies demonstrate that complement activation contributes to the skeletal muscle pathology and dysfunction in PDAC, suggesting that the complement system may possess therapeutic potential in preserving skeletal muscle mass and function.

## Abstract 2

Complement system activation plays a major role in pancreatic cancer cachexia, which was informed from proteomic analysis of skeletal muscle from pancreatic cancer patients.

## Introduction

Many advanced-stage cancer patients suffer from involuntary loss of body weight due to loss of skeletal muscle mass, and often an accompanying loss of fat mass — a condition known as cachexia (1). The loss of skeletal muscle mass is associated with impairments in physical function, compromising activities of daily living, independence, and quality of life; limits, or even precludes, treatment options; and is strongly predictive of early mortality (2). There is therefore a dire need for interventions to prevent or reverse cachexia in cancer patients. However, to date, the only therapy approved is in Japan, where anamorelin is approved as an appetite enhancer to counter anorexia in cachectic patients (3). One possible explanation for the current lack of therapeutics is the limited understanding of the underlying biological processes associated with muscle wasting and dysfunction in muscle of cachectic cancer patients. Without such knowledge, the successful translation of potential therapeutics may be more difficult. The more that can be learned about muscle biology and pathology from cachectic cancer patients, the better positioned therapeutics will be to target processes with established translational relevance.

In the current study, we therefore conducted unbiased total proteome profiling using multiplexed sample labeling tandem mass tags (TMTs) and liquid chromatography–tandem mass spectrometry (LC-MS/MS) to identify proteins differentially expressed in the skeletal muscle of cachectic pancreatic cancer patients compared with non-cancer controls. We selected patients with pancreatic cancer to study because cachexia is highly prevalent in such patients, present in more than 60% of newly diagnosed patients and increasing to more than 80% as the disease progresses (4). We subsequently used immunohistochemical (IHC) analyses to substantiate a subset of findings from the proteome dataset using tissue sections taken from the same, and additional, cancer patients. These data identified an enrichment of complement proteins and activation of immune system processes in muscles from cachectic pancreatic ductal adenocarcinoma (PDAC) patients. Although the complement system is an essential component of the host response to infection and cellular injury, playing key roles in pathogen identification, opsonization, and activation of both innate and adaptive immune cells (5), inappropriate or excessive complement activation can damage host tissues and promote inflammation (6, 7). In this regard, excessive complement activation has been implicated in the pathogenesis of various muscle diseases, including dysferlinopathy, polymyositis, dermatomyositis, and inclusion body myositis (8–10). Thus, we subsequently tested the role of complement activation in cancer-induced muscle pathologies by injecting KPC pancreatic cancer cells into the pancreas of wild-type mice or mice null for the central component of complement 3 (C3). Our data support the complement system as a key mediator of PDAC-induced cachexia that facilitates pathological remodeling and dysfunction of respiratory muscles, including immune cell infiltration, myofiber atrophy, and fibrotic tissue remodeling.

## Results

### Patients.

Demographics and clinicopathological details for patients included in the proteomic analysis are presented in Table 1. All patients included in the current study were female, and all PDAC patients were naive to neoadjuvant therapy at the time of surgery. In accordance with recently published guidelines for the management of cancer cachexia (11), all PDAC patients were defined as cachectic, demonstrating involuntary body mass loss of greater than 5% and skeletal muscle depletion determined through CT-based measurements of skeletal muscle index, as previously described (12). In addition, the severity of cancer cachexia (grades 0–4) was determined using recently established, mortality-based criteria (13). In cases in which there was insufficient sample remaining from a patient for follow-up histological analyses after the proteomics, additional samples from non-cancer control (*n* = 3) and PDAC (*n* = 2) patients, which were not included in the initial proteomic analysis, were added. All additional patients were female and met the same clinical (naive to neoadjuvant therapy) and cachexia criteria as patients selected for proteomic analysis. Demographic and clinicopathological details for the expanded patient population are presented in Supplemental Table 1 (supplemental material available online with this article; https://doi.org/10.1172/JCI178806DS1).

### Pancreatic cancer cachexia alters skeletal muscle proteome.

**Figure 1:**
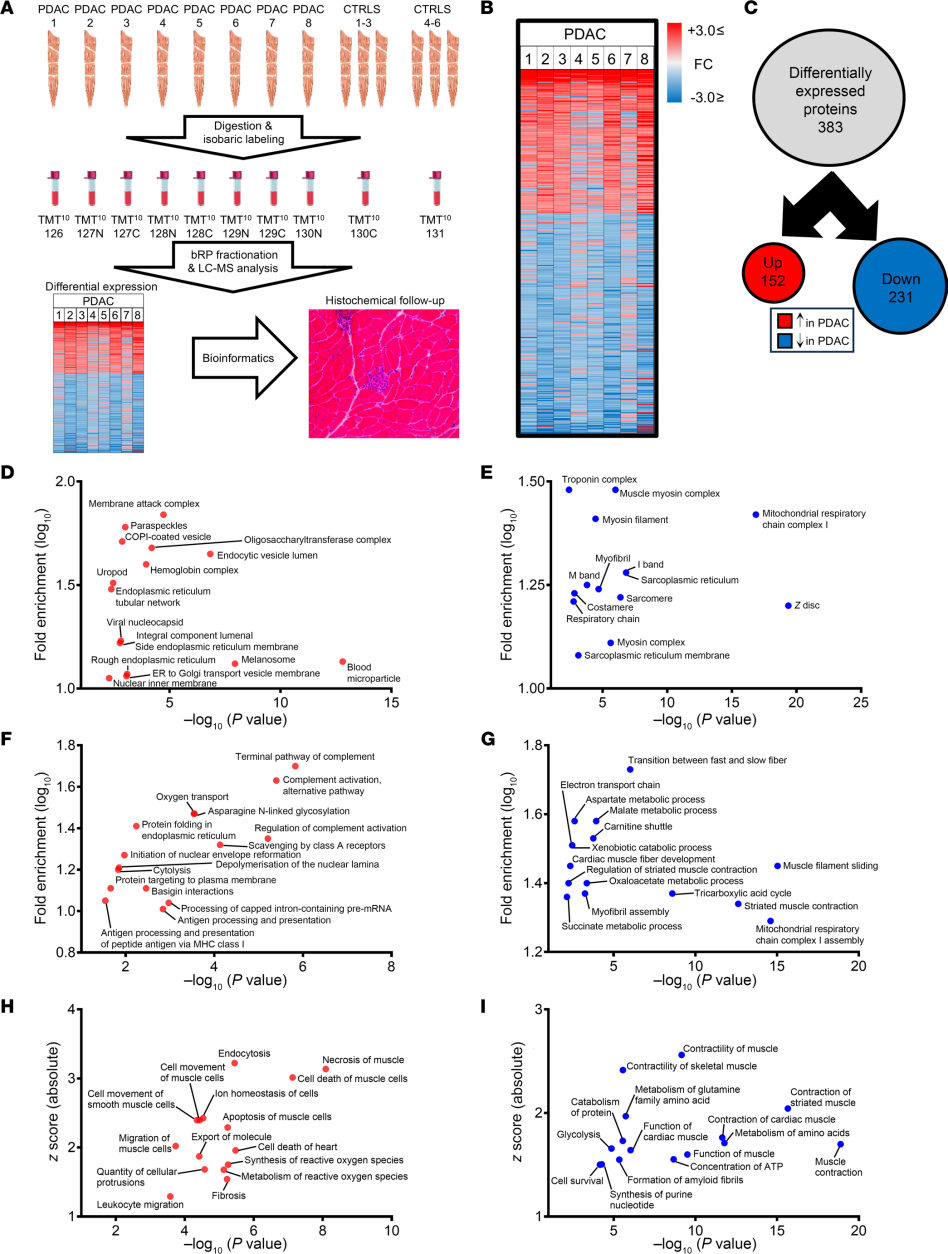
Skeletal muscle proteome is dysregulated in cachectic PDAC patients. (**A**) Rectus abdominis biopsies acquired from cachectic PDAC patients (*n* = 8) and non-cancer controls (CTRL; *n* = 6) were subjected to 10-plex tandem mass tag proteomics. Biopsies were enzymatically digested to isolate peptides, which were labeled with 10 distinct isobaric tags (8 PDAC plus 2 pooled CTRL [*n* = 6 total]), and combined for liquid chromatography tandem mass spectrometry analysis. Differentially expressed proteins (DEPs) between PDAC and CTRL were analyzed through a bioinformatics pipeline, followed by IHC analyses of skeletal muscle cross sections. (**B**) Heatmap displays the relative expression (vs. CTRL) of proteins that met differential expression criteria (≥2 peptides; –1.25 ≥ median fold change ≥ 1.25; FDR-adjusted *P* value ≤ 0.05) between PDAC and CTRL. (**C**) Among the DEPs (*n* = 383) in PDAC skeletal muscle, most are downregulated relative to CTRL. (**D** and **E**) The top 15 cellular components enriched by upregulated (**D**) and downregulated (**E**) proteins were identified through DAVID Functional Annotation. (**F** and **G**) The top 15 biological processes enriched by upregulated (**F**) and downregulated (**G**) proteins were identified through DAVID Function Annotations, Kyoto Encyclopedia of Genes and Genomes, and Reactome pathway databases. (**H** and **I**) The top 15 activated (**H**) and inhibited (**I**) functions were identified through IPA analysis of up- and downregulated proteins, combined. Annotations with fewer than 3 proteins or a *P* value greater than 0.05 were excluded.

The initial goal of this study was to characterize the skeletal muscle proteome in cachectic PDAC patients to better understand the mechanisms associated with cachexia in this population. To achieve this goal, we collected skeletal muscle biopsies of the rectus abdominis (*n* = 14; Table 1). Soluble peptides from cachectic PDAC patients (*n* = 8) and weight-stable non-cancer controls (CTRL; *n* = 6), separated into 2 pooled CTRL groups (*n* = 3 pooled samples per group), were labeled with one of 10 distinct isobaric TMT labels, and annotated proteins were compared between PDAC and CTRL (Figure 1A). Following LC-MS/MS analysis, 61,463 peptides were detected among all samples, of which 44,560 peptides were uniquely identified. In total, 3,809 proteins were identified (1% FDR), of which 2,919 proteins annotated to at least 2 distinctly detected peptides. Using criteria outlined in Methods, 383 proteins were identified to be differentially expressed in the skeletal muscle of PDAC versus CTRL (Figure 1B and Supplemental Data File 1), of which 152 (39.7%) were upregulated and 231 (60.3%) were downregulated in the skeletal muscle of PDAC versus CTRL (Figure 1C).

To identify altered biological processes and pathways within the skeletal muscle of cachectic PDAC patients, differentially expressed proteins (DEPs) were analyzed using several bioinformatic platforms. Up- and downregulated proteins were analyzed separately to determine altered cellular components (Figure 1, D and E) and biological processes (Figure 1, F and G). The top cellular component terms enriched by proteins upregulated in muscle from PDAC patients included the membrane attack complex of the complement system, protein transport vesicles, blood microparticles, and various components of the endoplasmic reticulum (Figure 1D). Cellular component terms enriched by proteins downregulated in muscle from PDAC patients included various aspects of the sarcomere, including M band, I band, Z disc, muscle myosin complex, myosin filament, and troponin complex; the costamere; and mitochondrial respiratory chain complex I (Figure 1E). Further clustering of cellular components, which condenses proteins into organized classes of proteins, to identify cellular compartments of DEPs revealed that of the 152 DEPs that were increased, 84 annotated to “extracellular region,” 39 to “endoplasmic reticulum,” 33 to “cell junction,” 30 to “secretory vesicle,” and 18 to “blood microparticle.” When the 231 downregulated DEPs were clustered, 87 proteins annotated to “mitochondrion,” 40 to “myofibril,” 14 to “sarcoplasm,” 12 to “sarcolemma,” and 10 to “myosin complex” (Supplemental Data File 1).

Biological processes enriched by proteins upregulated in muscle from PDAC patients included multiple pathways of the complement system (terminal pathway of complement, alternative pathway of complement activation, regulation of complement activation), oxygen transport, protein folding in endoplasmic reticulum, and antigen presentation and processing of peptide via MHC class I (Figure 1F). Proteins downregulated in muscle from PDAC patients showed enrichment of biological processes related to transition between fast and slow fiber, multiple aspects of muscle contraction (muscle filament sliding, regulation of striated muscle contraction, striated muscle contraction), mitochondrial processes (mitochondrial respiratory chain complex I assembly, tricarboxylic acid cycle, and electron transport chain), and metabolic processes (aspartate metabolism, malate metabolism, oxaloacetate metabolism, succinate metabolism) (Figure 1G). Up- and downregulated proteins were analyzed together via Ingenuity Pathway Analysis (IPA) to predict activated (Figure 1H) and inhibited (Figure 1I) diseases and functions in PDAC skeletal muscle. Activated functions in PDAC skeletal muscle included several aspects of muscle cell death (necrosis of muscle, cell death of muscle cells, apoptosis of muscle cells, cell death of heart), movement/migration of various cell types (muscle cells, smooth muscle cells, leukocytes), synthesis and metabolism of reactive oxygen species, and fibrosis. Functions predicted as inhibited in the skeletal muscle of PDAC predominantly included aspects of muscle contraction, as well as metabolism of amino acids, concentration of ATP, glycolysis, and cell survival.

### Cachectic PDAC patients exhibit muscle fiber atrophy and collagen remodeling.

**Figure 2:**
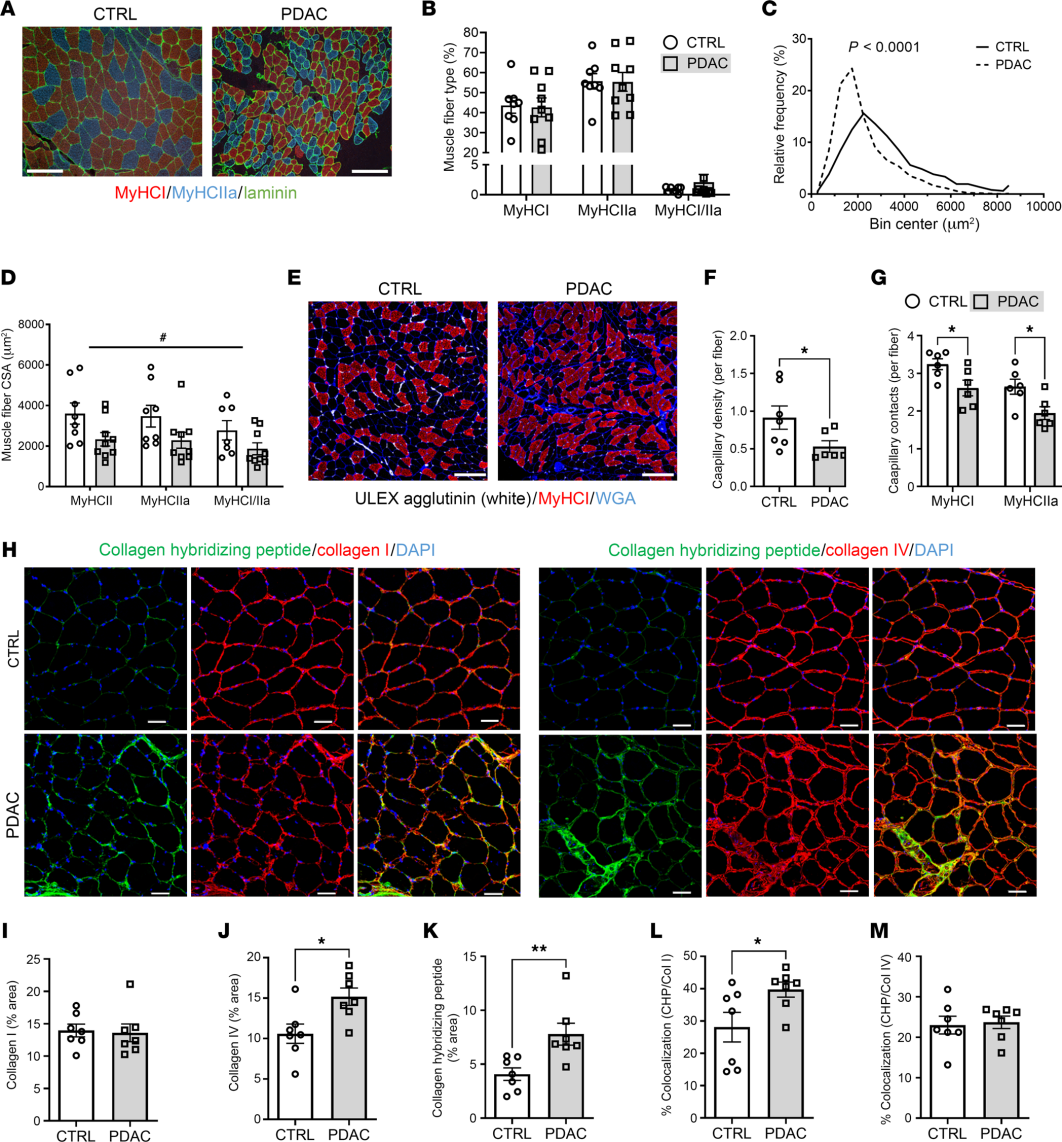
Skeletal muscle of cachectic PDAC patients exhibits worsened morphology and remodeling of extracellular matrix. (**A**) Representative images of rectus abdominis cross sections from CTRL and PDAC stained for myosin heavy chain I (MyHC I; red), MyHC IIa (blue), and laminin (green); scale bars: 200 μm. (**B**) Relative abundance of fibers positive for each MyHC isoform in CTRL and PDAC. (**C**) Quantification of overall muscle fiber cross-sectional area (CSA) demonstrates a leftward shift in the proportion of small fibers in PDAC versus CTRL. Significance was assessed through a Gaussian least-squares regression of binned CSA data and calculation of the extra sum-of-squares *F* test. (**D**) Fiber type–specific CSA was also quantified. (**E**) Representative images of skeletal muscle cross sections stained for endothelial cells (*Ulex europaeus* agglutinin, white), MyHC I (red), and wheat germ agglutinin (WGA; blue); scale bars: 200 μm. (**F** and **G**) Capillary density (**F**) and fiber type–specific capillary contacts (**G**) were quantified in CTRL and PDAC. (**H**) Representative images of skeletal muscle cross sections stained for collagen hybridizing peptide (CHP; green), collagen I or IV (red), and DAPI (blue); scale bars: 50 μm. (**I**–**M**) Quantification of percentage muscle area positive for collagen I (**I**), collagen IV (**J**), and CHP (**K**), as well as quantitative colocalization analyses on cross sections stained for CHP and collagen I (**L**) and CHP and collagen IV (**M**). Data are presented as mean ± SEM, with individual data superimposed. Data are representative of *n* = 7–8 for CTRL and *n* = 6–9 for PDAC. Differences were assessed using a 2-way ANOVA with Šidák’s post hoc analysis (**D** and **G**), Mann-Whitney *U* test (**F**), and Student’s 2-tailed *t* test (**J**–**L**). ^#^*P* < 0.05 main effect of group, *P < 0.05, **P < 0.01.

We subsequently performed follow-up analyses using cross sections of rectus abdominis from CTRL and PDAC patients to assess muscle morphology and specific biological pathways identified through proteomic analyses as dysregulated in PDAC muscle. Despite our findings of dysregulated proteins involved in “transition between fast and slow fiber” in PDAC muscle, fiber-type analyses revealed no differences in the relative abundances of myosin heavy chain (MyHC) I, IIa, and I/IIa hybrid fibers between PDAC and CTRL (Figure 2, A and B). However, upon quantification of overall muscle fiber cross-sectional area (CSA), skeletal muscle from PDAC patients exhibited a significant leftward shift toward a greater proportion of smaller muscle fibers (Figure 2C). Similarly, the CSA of each fiber type was reduced in PDAC patients versus CTRL (Figure 2D), where MyHC I, IIa, and I/IIa hybrid fibers were about 35%, about 34%, and about 33% smaller, respectively. In addition to muscle fiber atrophy, staining of muscle cross sections with *Ulex europaeus* agglutinin-1 (UEA1) to identify endothelial cells (14) revealed an approximately 42% reduction in capillary density (total capillaries/total fibers) in PDAC versus CTRL (Figure 2, E and F). This is in close agreement with recent findings from Kim et al., who also used UEA1 and found a decrease in muscle vascular density in cachectic cancer patients (15). To determine whether capillary regression occurred in a fiber type–specific manner, capillary contacts were quantified for individual MyHC I and MyHC IIa fibers. Similarly to total capillary density, capillary contacts were reduced for MyHC I (~19%) and MyHC IIa (~27%) fibers in PDAC versus CTRL (Figure 2G).

In support of our previous finding of increased skeletal muscle area occupied by collagen in the rectus abdominis of cachectic PDAC patients (16), the unbiased proteomic analysis in the current study identified several collagen proteins as differentially expressed in the skeletal muscle of cachectic PDAC versus CTRL (Supplemental Data File 1). PDAC muscle showed reductions in the fibrillar type I collagens COL1A1 (–2.1-fold, adjusted *P* value [*P*_adj_] = 0.005) and COL1A2 (–1.9-fold, *P*_adj_ = 2.6 × 10^–5^), in combination with reduced COL12A1 (–2.2-fold, *P*_adj_ = 1.2 × 10^–6^), which interacts with collagen type I and is important for muscle integrity (17). In contrast, collagen type IV, the most abundantly expressed collagen of the basement membrane in skeletal muscle tissue (18), was increased in PDAC muscle, including COL4A1 (1.4-fold, *P*_adj_ = 6.1 × 10^–5^) and COL4A2 (1.4-fold, *P*_adj_ = 1.5 × 10^–5^). To further investigate these findings and the degree of collagen remodeling, muscle cross sections were stained for collagen type I, collagen type IV, and collagen hybridizing peptide (CHP), a key peptide that binds only to remodeling/unfolded collagen (19) or damaged collagen fibrils (20) (Figure 2H). While the muscle area occupied by collagen type I was not different between PDAC and CTRL, the area occupied by collagen type IV was approximately 30% greater in PDAC compared with CTRL (Figure 2, I and J). Moreover, binding of CHP was approximately 48% greater in PDAC compared with CTRL (Figure 2K), suggesting increased remodeling and/or damage to existing collagen. To determine whether collagen remodeling/damage differed between collagen types, colocalization analyses were performed between CHP and collagen type I or collagen type IV. Colocalization between CHP and collagen type I was approximately 29% greater in PDAC versus CTRL (Figure 2L), whereas colocalization between CHP and collagen type IV was not different between groups (Figure 2M). These combined findings suggest substantial extracellular matrix (ECM) remodeling in the skeletal muscle of cachectic PDAC patients, characterized by increased remodeling/damage of collagen type I, and increased abundance of collagen type IV.

### Complement activation in skeletal muscle of cachectic PDAC patients.

**Figure 3:**
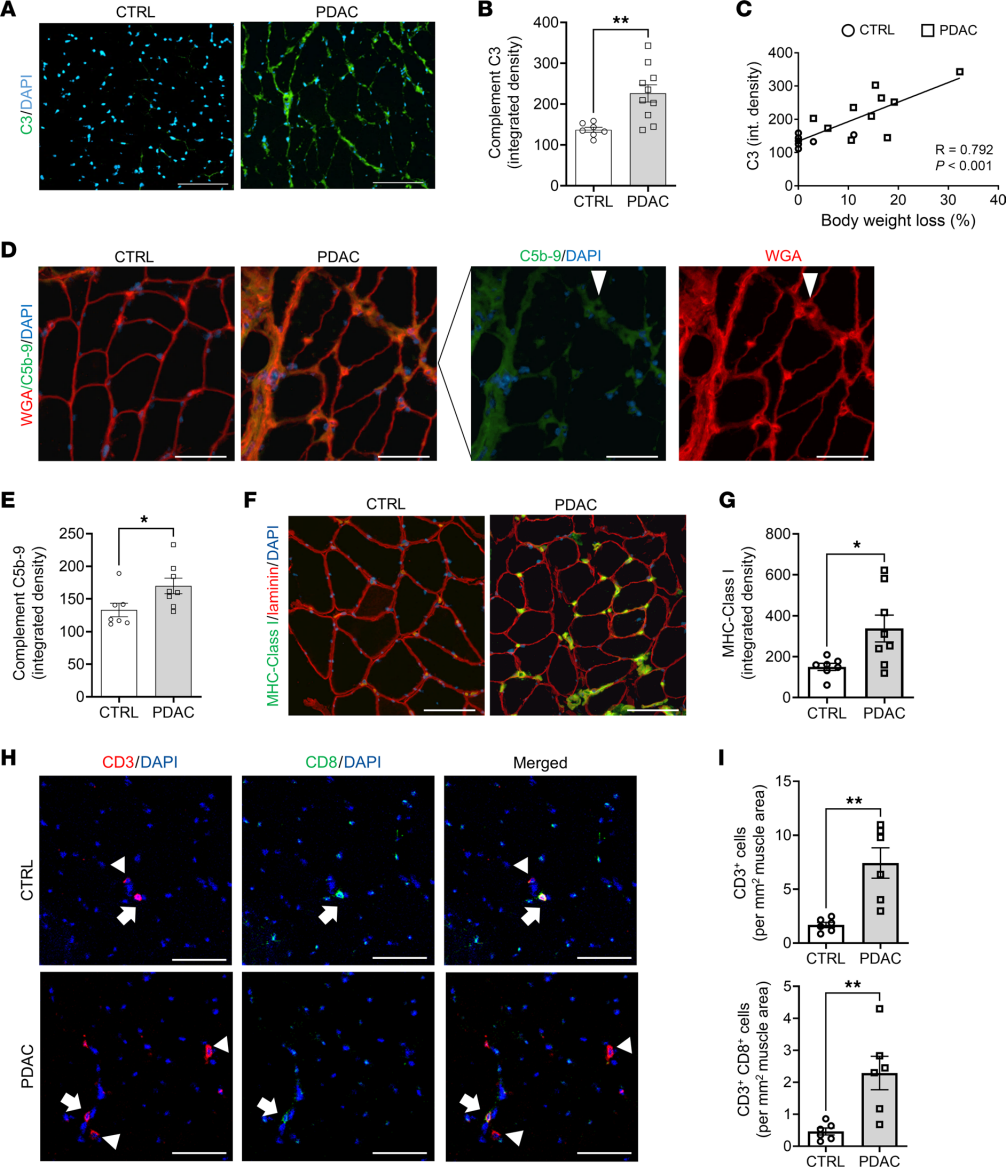
Complement activation in skeletal muscle of cachectic PDAC patients. (**A** and **B**) Representative images (**A**) and quantification (**B**) of rectus abdominis cross sections from CTRL and PDAC stained for complement component C3 (green); scale bars: 100 μm. (**C**) Correlation between complement C3 deposition and body weight loss. (**D** and **E**) Representative images (**D**) and quantification (**E**) of formation of terminal complement complex C5b-9 (membrane attack complex, green; arrowheads show circumferential staining of endomysial capillaries) in skeletal muscle of CTRL and PDAC; scale bars: 100 μm. (**F** and **G**) Representative images (**F**) and quantification (**G**) of muscle MHC-I abundance in skeletal muscle of CTRL and PDAC; scale bars: 100 μm. (**H**) Representative images of skeletal muscle stained for markers of total T cells (CD3, red, arrowheads) and cytotoxic T cells (CD8, green, arrows); scale bars: 50 μm. (**I**) Quantification of total infiltrating T cells and infiltrating cytotoxic T cells was performed in CTRL and PDAC. Data are presented as mean ± SEM, with individual data superimposed. Data are representative of *n* = 6–7 for CTRL and *n* = 6–10 for PDAC. Differences were assessed using Student’s 2-tailed *t* test (**B** and **G**), linear regression analysis (**C**), and Mann-Whitney *U* test (**E** and **I**). *P < 0.05, **P < 0.01.

Various biological processes could lead to skeletal muscle ECM remodeling in PDAC patients, including inflammation. In this regard, the skeletal muscle proteome revealed several blood components to be elevated in muscles of cachectic PDAC patients, including albumin and other plasma proteins, which are known to exit the vasculature through leaky capillaries during states of tissue and/or systemic inflammation. Of the plasma proteins increased in PDAC skeletal muscle, proteins of the complement system, which is involved in both innate and adaptive immune responses, were highly enriched. We therefore further evaluated local indices of complement activation in PDAC muscle. To do this we first immunohistochemically analyzed skeletal muscle cross sections for complement component 3 (C3) — the central component of complement system on which the 3 canonical pathways of complement activation converge. Compared with CTRL, skeletal muscle of PDAC patients showed an approximately 65% increase in C3 present in the muscle interstitium (Figure 3, A and B). Moreover, C3 deposition was associated with a greater severity of cachexia, as demonstrated by a positive correlation with body weight loss (Figure 3C). Of the 7 complement system proteins identified in the proteomics as being differentially expressed between CTRL and PDAC (Supplemental Data File 1), four are members of the terminal complement pathway, which upon activation forms the protein complex C5b-9, also known as the membrane attack complex (MAC). We therefore assessed MAC deposition in skeletal muscle cross sections through IHC labeling of C5b-9 (Figure 3D). Relative to CTRL, C5b-9 deposition was approximately 27% greater in the skeletal muscle of PDAC patients (Figure 3E). Notably, the staining pattern of C5b-9 in PDAC muscle revealed strong circumferential staining of endomysial capillaries, comparable to that observed in patients with dermatomyositis (21). In dermatomyositis, microvascular complement deposition is a suspected mechanism contributing to skeletal muscle capillary damage, inflammation, and loss of capillary density (10, 21), suggesting that a similar mechanism could be at play in muscles of cachectic PDAC patients.

In addition to complement activation, we observed additional immune-related processes elevated in PDAC muscle, including “antigen presentation and processing via MHC class I.” The major histocompatibility complex class I (MHC-I) antigen presentation pathway is upregulated during innate immune responses and plays an important role in presenting intracellular antigens (peptide fragments) to CD8^+^ cytotoxic T cells. When the antigen displayed by MHC-I on the surface of a cell is recognized as foreign (i.e., derived from pathogens, cancer cells, or abnormal cells) by CD8^+^ T cells with specificity for that antigen, this triggers CD8^+^ T cell responses that induce cell death. While MHC-I is typically expressed at very low levels by mature muscle cells, its upregulation in skeletal muscle is a common feature of various myopathies in which patients experience inflammation and skeletal muscle weakness (22–24). Moreover, studies have further shown that conditional upregulation of MHC-I in skeletal muscle is sufficient to drive inflammatory myositis in mice (25). We identified several components of MHC-I to be upregulated in the skeletal muscle of PDAC versus CTRL (Supplemental Data File 1), including HLA class I histocompatibility antigen, A-2 α chain (HLA-A; 1.8-fold, *P*_adj_ = 0.03), calreticulin (CALR; 1.5-fold, *P*_adj_ = 0.001), calnexin (CALX; 1.4-fold, *P*_adj_ = 0.0001), and β_2_-microglobulin (B2M; 1.8-fold, *P*_adj_ = 0.06). In support of these findings, IHC assessment of MHC-I in muscle cross sections revealed a 2.25-fold increased abundance in skeletal muscle of PDAC patients (Figure 3, F and G). While most of the MHC-I immunoreactivity localized to cells in the interstitium, some PDAC patients also exhibited sarcolemmal staining associated with myofibers. Since a primary function of MHC-I is to present endogenous antigens on the cell surface to CD8^+^ cytotoxic T cells (26), we hypothesized that skeletal muscle of PDAC patients may also show increased infiltration of such cells. In support of this, IHC labeling of CD3, as a T cell marker, and CD8, as a marker of cytotoxic T cells, revealed a significant 5-fold increase in cytotoxic T cells (CD3^+^, CD8^+^) in PDAC muscle cross sections compared with CTRL (Figure 3, H and I).

### The mouse diaphragm recapitulates complement activation and fibrotic remodeling in a murine model of PDAC.

**Figure 4:**
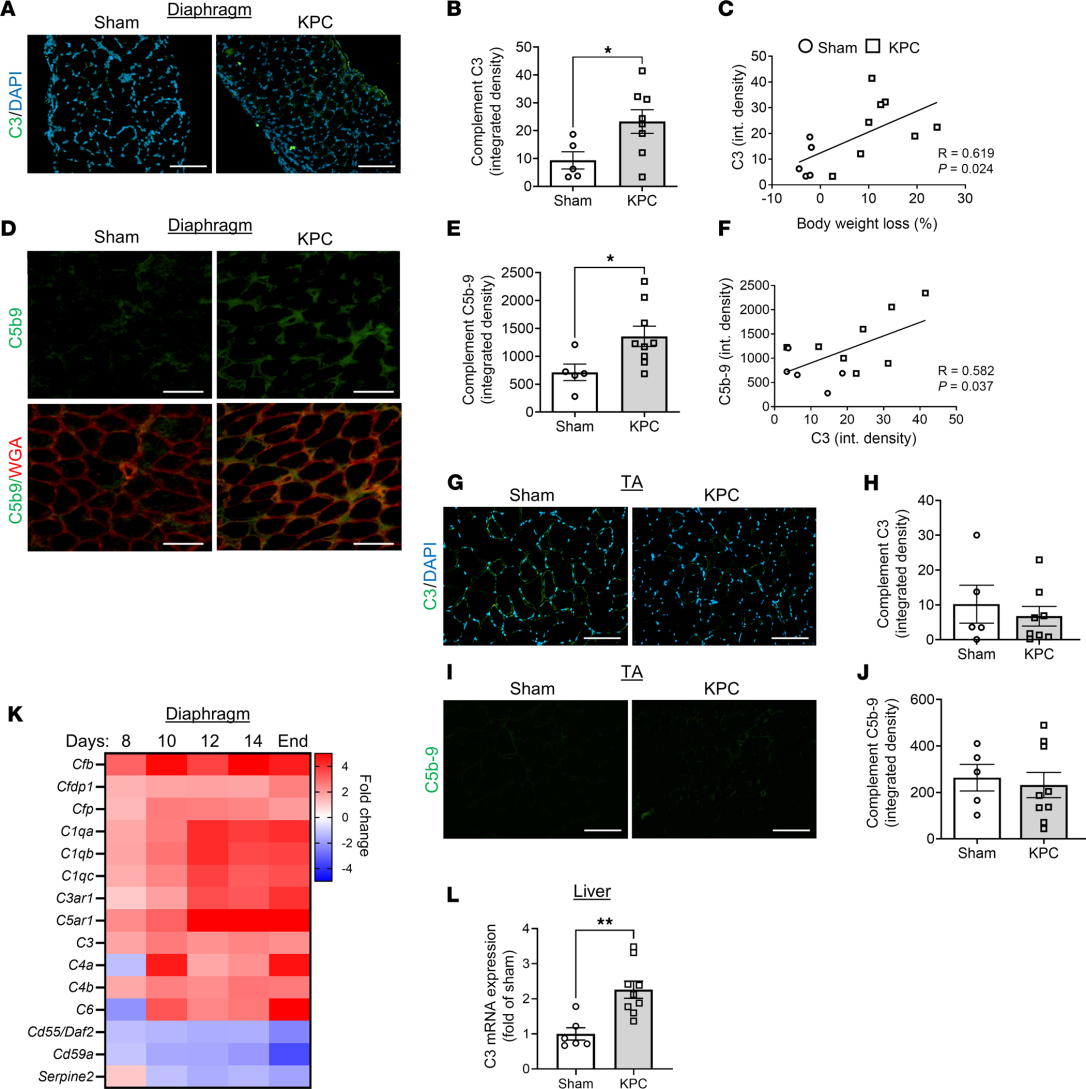
Orthotopic KPC model of PDAC-associated cachexia recapitulates skeletal muscle complement activation observed in cachectic PDAC patients. (**A** and **B**) Representative images (**A**) and quantification (**B**) of costal diaphragm stained for complement component C3 (green) and DAPI (blue); scale bars: 100 μm. (**C**) Correlation between complement C3 deposition in diaphragm and body weight loss. (**D** and **E**) Representative images (**D**) and quantification (**E**) of costal diaphragm stained for terminal complement complex C5b-9 (membrane attack complex, green) and wheat germ agglutinin (red); scale bars: 50 μm. (**F**) Correlation between complement C3 deposition and C5b-9 formation in costal diaphragm. (**G** and **H**) Representative images (**G**) and quantification (**H**) of TA stained for complement component C3 (green) and DAPI (blue); scale bars: 100 μm. (**I** and **J**) Representative images (**I**) and quantification (**J**) of TA stained for terminal complement complex C5b-9 (membrane attack complex, green); scale bars: 100 μm. (**K**) Heatmap depicting changes in the expression level of complement transcripts in the diaphragm muscles of mice with orthotopic KPC tumors during cachexia progression relative to control mice, extracted from previously published RNA-Seq data by Neyroud et al. (32). (**L**) Quantitative reverse transcriptase PCR analysis of complement C3 mRNA expression from liver of sham and KPC tumor–bearing mice. Data are presented as mean ± SEM, with individual data superimposed. Data are representative of *n* = 5–9 mice per group. Differences were assessed using Student’s 2-tailed *t* test (**B**, **E**, and **L**) and linear regression analysis (**C** and **F**). *P < 0.05, **P < 0.01.

Combined, our proteomic studies and follow-up IHC studies implicate skeletal muscle microvascular depletion and local tissue inflammation as key events that may contribute to pathological remodeling and wasting of skeletal muscles in PDAC patients exhibiting cachexia. Although the initiating triggers of these events are not entirely clear, our data suggest that the complement system may be involved. In this regard, although transient activation of the complement system has been established to facilitate skeletal muscle regeneration following acute injury through the recruitment of monocytes/macrophages that support the regenerative process (27, 28), inappropriate or uncontrolled activation of complement can amplify inflammation and contribute to tissue injury and pathological remodeling (8, 29, 30). To build on these findings and test whether the complement system plays a causative role in mediating cancer-induced skeletal muscle pathologies, we used the KPC preclinical mouse model of PDAC. In this model, KPC cells isolated from the tumor of a KPC mouse are injected into the mouse pancreas, which induces key features of PDAC-associated cachexia including myofiber atrophy and weakness (31), and pathological remodeling in the diaphragm consistent with that of PDAC patients, including infiltration of leukocytes, expansion of platelet-derived growth factor receptor α–positive (PDGFRα^+^) mesenchymal progenitors, and collagen deposition (32). As with PDAC patients, we found that the diaphragm muscles of KPC mice showed increased C3 deposition (Figure 4, A and B) that positively correlated with body weight loss (Figure 4C), and increased formation of MAC/C5b-9 complexes, including around endomysial capillaries (Figure 4, D and E). Unsurprisingly, C3 deposition and MAC/C5b-9 formation were tightly correlated (Figure 4F). In contrast, the tibialis anterior (TA) muscle did not show any increase in C3 or C5b-9 staining (Figure 4, G–J), supporting our previous findings that skeletal muscles more proximal to PDAC tumors, such as the diaphragm and the rectus abdominis muscle, show more pronounced activation of inflammatory processes and pathological remodeling compared with peripheral locomotor muscles (31–33). Through extraction of complement genes from our recently published RNA-Seq dataset (32), we further show that diaphragm muscles of KPC mice exhibited upregulated transcript levels for multiple complement components and a repression in transcripts for complement inhibitors at all time points on the cachexia continuum (Figure 4K). Collectively, these data support an increase in complement pathway activation in muscles proximal to PDAC tumors in mice and people exhibiting cachexia, and suggest the involvement of *local* complement production in muscle tissue, which could be complementary to any systemic complement that may exit the vasculature and enter the muscle tissue. To assess systemic production of complement, we also measured C3 mRNA levels in the liver, which is the major site of plasma protein production, including complement (34), and found an increase in C3 mRNA (Figure 4L).

### Deletion of C3 attenuates ascites and wasting of muscle and fat in a murine model of PDAC-associated cachexia.

**Figure 5:**
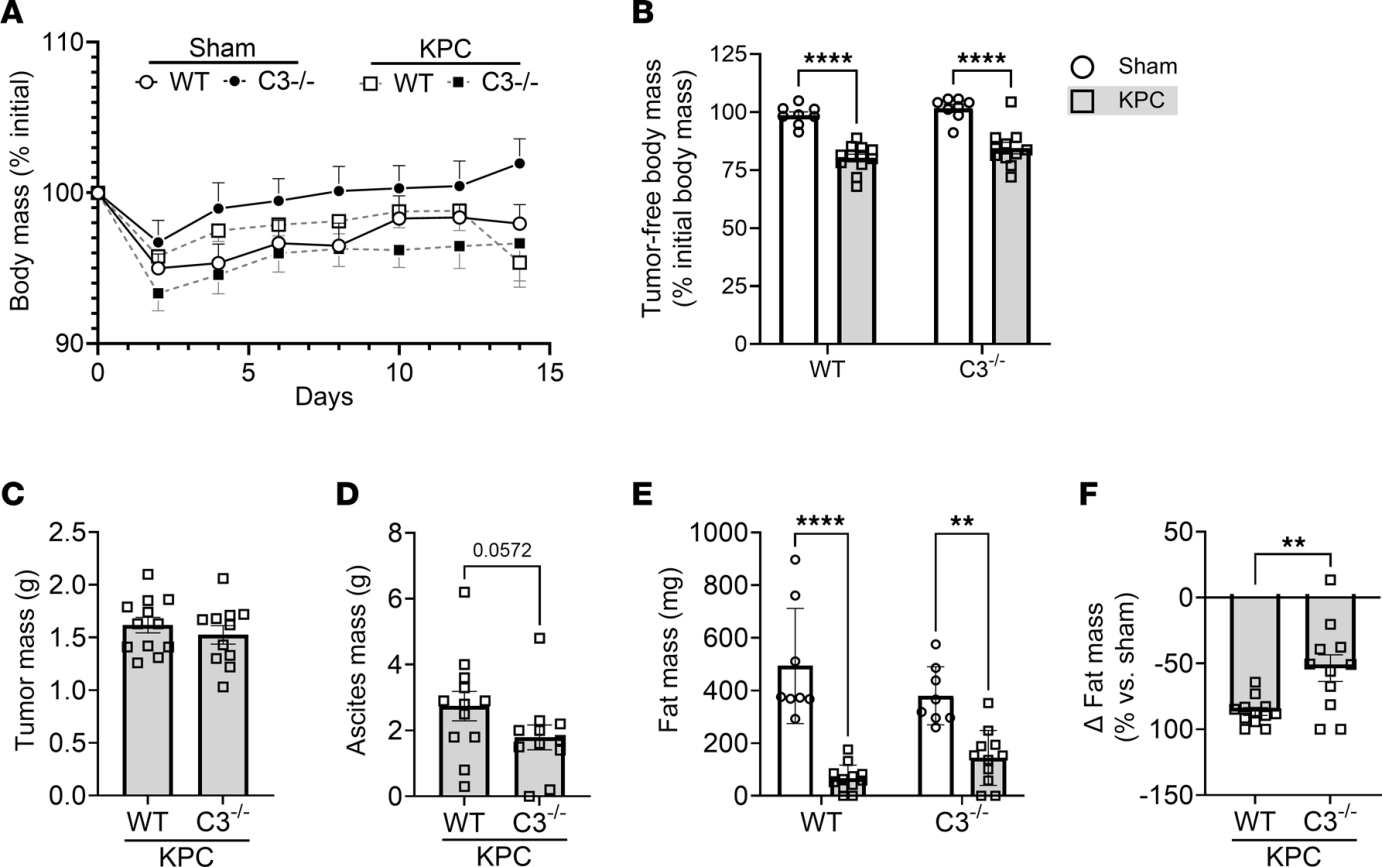
Deletion of C3 attenuates ascites and fat wasting in murine model of PDAC-associated cachexia. C57BL/6J wild-type (WT) mice and complement C3–null C57BL/6J mice (C3^–/–^) underwent orthotopic surgeries in which either PBS (sham) or KPC tumor cells were implanted into the pancreas. (**A**) Body mass was monitored over the course of tumor burden until IACUC-mandated endpoint (day 14). (**B**) At endpoint, tumor-free body mass was reduced in KPC tumor–bearing mice (squares) versus sham (circles). (**C**–**F**) Tumor mass (**C**), ascites mass (**D**), and fat mass (**E** and **F**) on day 14 after surgery. (**F**) Tumor-induced fat wasting relative to sham controls was determined within each genotype (WT, C3^–/–^). Data are presented as mean ± SEM, with individual data superimposed. Data are representative of *n* = 8–12 mice per group. Differences were assessed using a 2-way ANOVA with Šidák’s post hoc analysis (**B** and **E**), Mann-Whitney *U* test (**D**), and Student’s 2-tailed *t* test (**F**). **P < 0.01, ****P < 0.0001.

**Figure 6:**
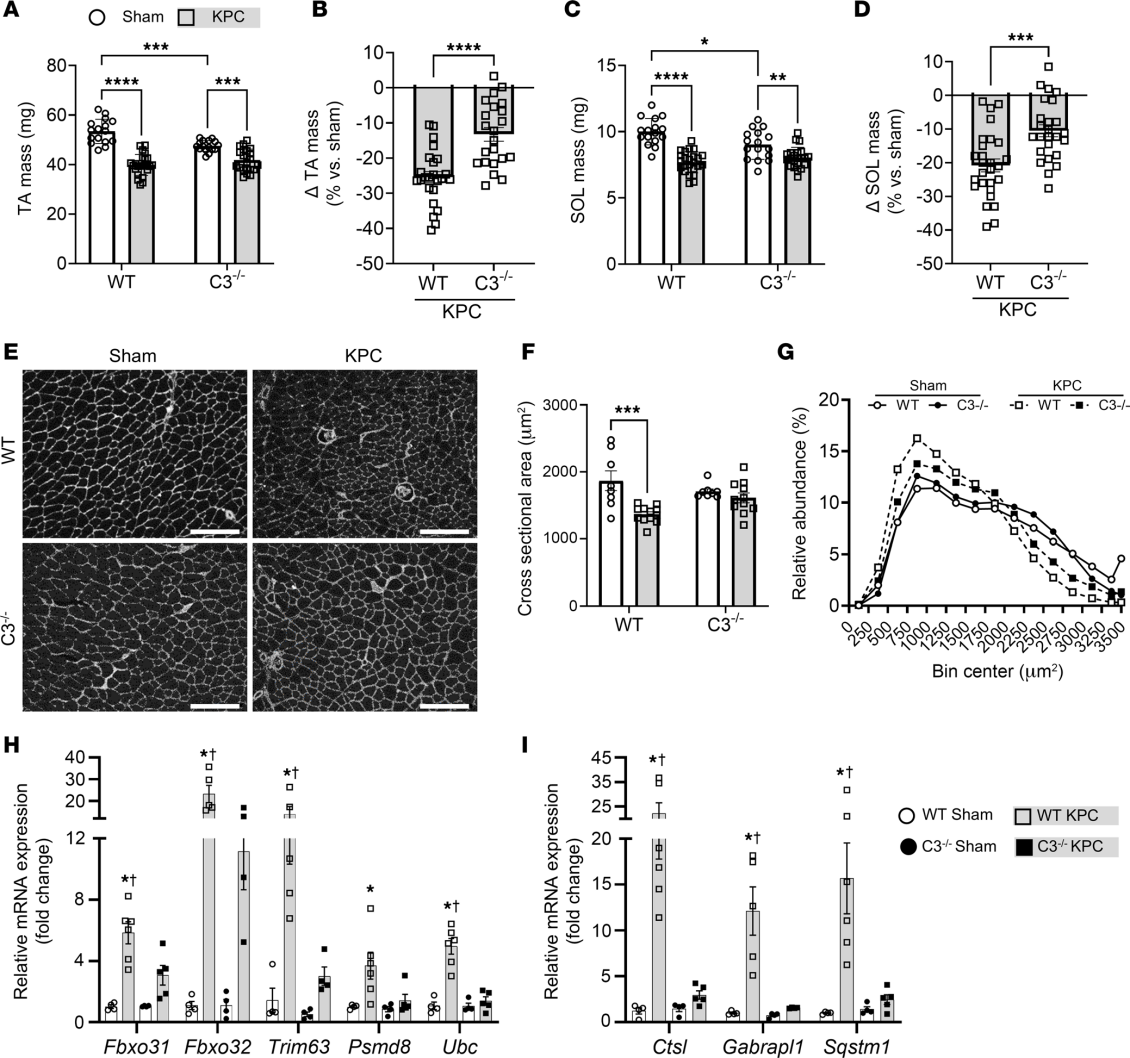
Deletion of C3 attenuates KPC-induced limb muscle wasting. (**A**) Tibialis anterior (TA) mass was reduced in KPC tumor–bearing mice (squares) versus sham (circles). (**B**) Deletion of complement component C3 attenuated TA muscle wasting. (**C** and **D**) Soleus (SOL) mass was reduced in KPC tumor–bearing mice (**C**); however, the deletion of C3 attenuated SOL wasting (**D**). (**E**) Representative images of TA cross sections stained for wheat germ agglutinin (white); scale bars: 200 μm. (**F** and **G**) Quantification of TA fiber cross-sectional area (CSA) (**F**) and distribution of fiber CSA (**G**). Data are representative of *n* = 8–12 mice per group. *P < 0.05, **P < 0.01, ***P < 0.001 ****P < 0.0001. (**H** and **I**) Quantitative reverse transcriptase PCR analysis of atrophy-related genes of interest involved in the ubiquitin-proteasome system (**H**) and the autophagy-lysosome system (**I**) in the TA; *n* = 4–6 mice per group. *P < 0.05 vs. WT sham group. ^†^*P* < 0.05 vs. C3^–/–^ KPC group. Data are presented as mean ± SEM, with individual data superimposed. Differences were assessed using a 2-way ANOVA with Šidák’s post hoc analysis (**A**, **C**, **F**, **H**, and **I**) and Student’s 2-tailed *t* test (**B** and **D**).

To test the role of the complement system in PDAC-associated wasting and pathological remodeling of skeletal muscle, we injected KPC cells into the pancreas of either wild-type (WT) mice or mice with genetic deletion of the central regulator of complement, C3 (C3^–/–^ mice). At study endpoint, body mass was significantly reduced in both WT and C3^–/–^ KPC mice relative to sham controls (Figure 5, A and B). Deletion of C3 from the murine host did not impact pancreatic tumor growth (Figure 5C) or substantially impact the tumor transcriptome, as determined via RNA-Seq of KPC tumors from WT and C3^–/–^ mice. Indeed, only 16 genes were differentially expressed between genotypes when *P*_adj_ less than 0.05 was used, which increased to only 26 genes when the criterion was loosened to *P*_adj_ less than 0.10 (Supplemental Data File 2). Moreover, upon extraction of previously identified marker genes that measure 14 immune cell populations (35), we found no significant differences in any of these marker genes between tumors from WT and C3^–/–^ mice (Supplemental Data File 2). These data therefore support a similar overall transcriptional signature, including that reflective of the immune landscape, in KPC tumors from WT and C3^–/–^ mice. It is important to note, however, that KPC cells injected into mice retain the C3 gene, and are thus capable of producing C3 locally within the tumor. C3 deletion from the murine host did, however, attenuate the development of peritoneal ascites (Figure 5D), a prognostic factor negatively associated with survival in PDAC patients (36). While epididymal fat mass was reduced in WT and C3^–/–^ KPC mice (Figure 5E), the deletion of C3 imparted a 38% reduction in fat wasting in comparison with WT mice (Figure 5F). In agreement with our previous findings (12), KPC tumors also induced wasting of the TA (Figure 6, A–D) and soleus (SOL; Figure 6, C and D); limb muscles which are phenotypically distinct from each other. In WT mice, muscle mass was reduced by approximately 28% in the TA and by approximately 20% in the SOL in response to KPC tumors. In contrast, in C3^–/–^ mice KPC tumors reduced muscle mass by about 12% in the TA and about 10% in the SOL, resulting in about 50% sparing of tumor-induced muscle loss. Since muscles of cancer-free C3^–/–^ mice were significantly smaller than those of cancer-free WT mice, we cannot rule out that lower starting muscle mass leads to a differential response to cachexia. However, further analysis of TA muscle fiber cross-sectional area (CSA) revealed no significant differences between cancer-free WT and C3^–/–^ mice. In contrast, KPC tumors elicited an approximately 27% reduction in TA fiber CSA (Figure 6, E and F) and a leftward shift in the fiber size distribution curve in WT mice (Figure 6G), which did not similarly occur in C3^–/–^ mice (Figure 6, F and G). Since transcriptional upregulation of components of the ubiquitin-proteasome and autophagy-lysosomal pathways involved in muscle protein degradation are well established to contribute to muscle wasting, including that induced by cancer (37), we measured transcript levels of several key markers of these pathways. A significant increase in the expression level of several markers of the ubiquitin-proteasome system (*Fbxo31*, *Fbxo32*/*Atrogin-1*, *Trim63*/*MuRF1*, *Psmd8*, and *Ubc*) and the autophagy-lysosome system (*Ctsl*, *Gabrapl1*, and *Sqstm1*) was observed in the muscles of WT, but not C3^–/–^, KPC mice (Figure 6, H and I). Together, these data implicate the complement system as a key mediator of PDAC-induced muscle wasting. However, since we did not find evidence of complement pathway activation in the TA (a locomotor muscle) of KPC mice, these findings suggest an indirect role of complement in mediating PDAC-induced muscle wasting, at least in a peripheral locomotor muscle.

### Deletion of C3 ameliorates pathological remodeling of the diaphragm in KPC mice.

**Figure 7:**
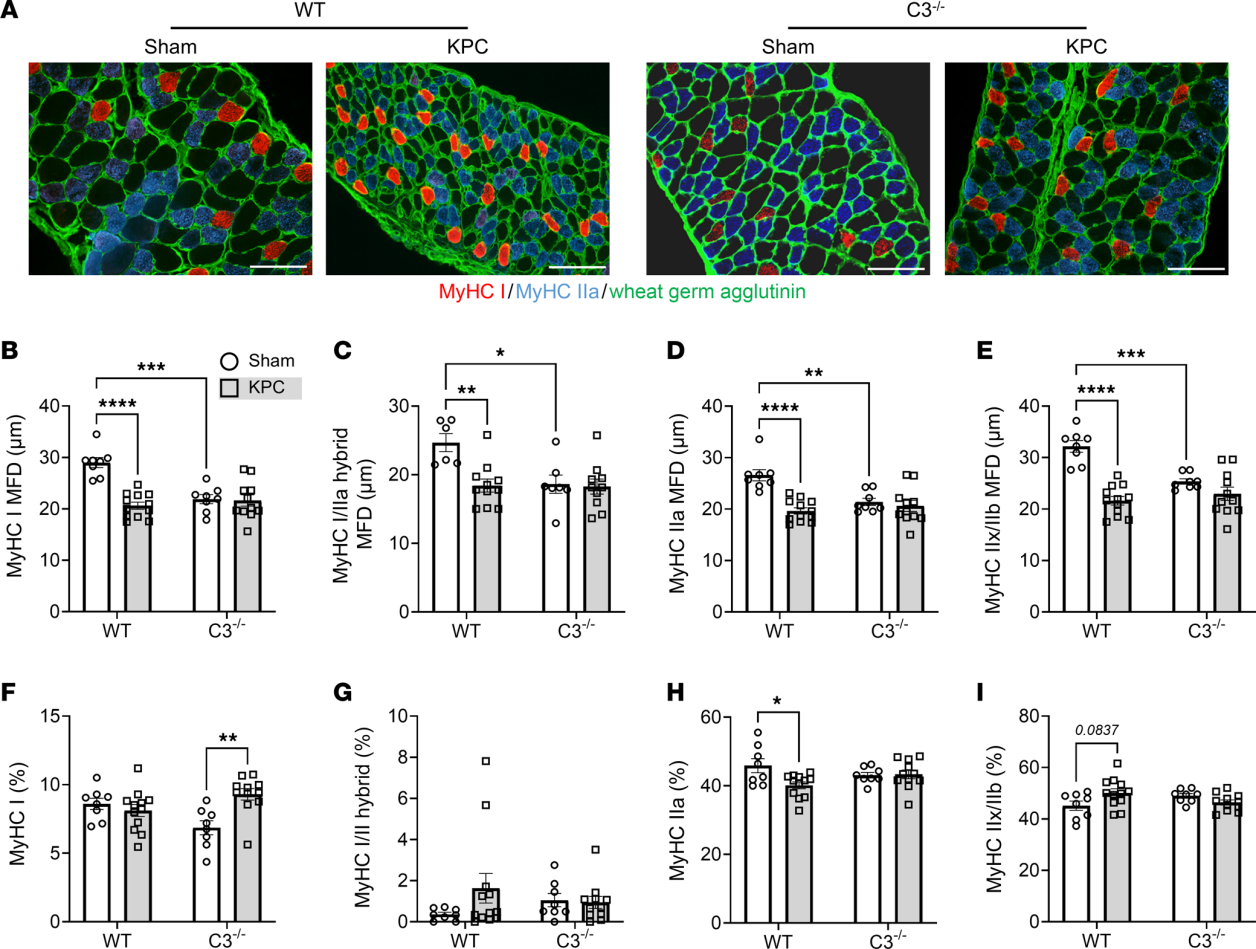
Deletion of C3 ameliorates KPC-induced diaphragm atrophy and fibrosis. (**A**) Representative images of costal diaphragm cross sections from sham and KPC tumor–bearing mice stained for MyHC I (red), MyHC IIa (blue), and wheat germ agglutinin (green); scale bars: 100 μm. (**B**–**E**) Fiber type–specific muscle fiber size, quantified as minimum Feret’s diameter (MFD), was determined for MyHC I fibers (**B**), MyHC I/IIa hybrid fibers (**C**), MyHC IIa fibers (**D**), and MyHC IIx/IIb (unstained) fibers (**E**). (**F**–**I**) Relative abundance of MyHC I (**F**), MyHC I/IIa hybrid (**G**), MyHC IIa (**H**), and MyHC IIx/IIb fibers (**I**). Data are presented as mean ± SEM, with individual data superimposed. Data are representative of *n* = 4–12 mice per group. Differences were assessed using a 2-way ANOVA with Šidák’s post hoc analysis. *P < 0.05, **P < 0.01, ***P < 0.001, ****P < 0.0001.

We subsequently determined the extent to which the complement system mediates KPC-induced wasting, inflammation, and pathological remodeling of the diaphragm, which, unlike locomotor muscles, shows evidence of complement pathway activation similar to that seen in rectus abdominis muscles from cachectic PDAC patients. Cross sections of costal diaphragm were subjected to IHC staining of MyHC I, MyHC IIa, and wheat germ agglutinin to assess fiber type–specific muscle fiber size (Figure 7, A–I). In WT mice, KPC tumors induced atrophy of MyHC I fibers (~29%; Figure 7B), MyHC I/IIa hybrid fibers (~26%; Figure 7C), MyHC IIa fibers (~26%; Figure 7D), and MyHC IIx/IIb fibers (~33%; Figure 7E). Similarly to locomotor muscles, diaphragm muscles from C3^–/–^ mice were protected from KPC-induced muscle fiber atrophy (Figure 7, B–E).

**Figure 8:**
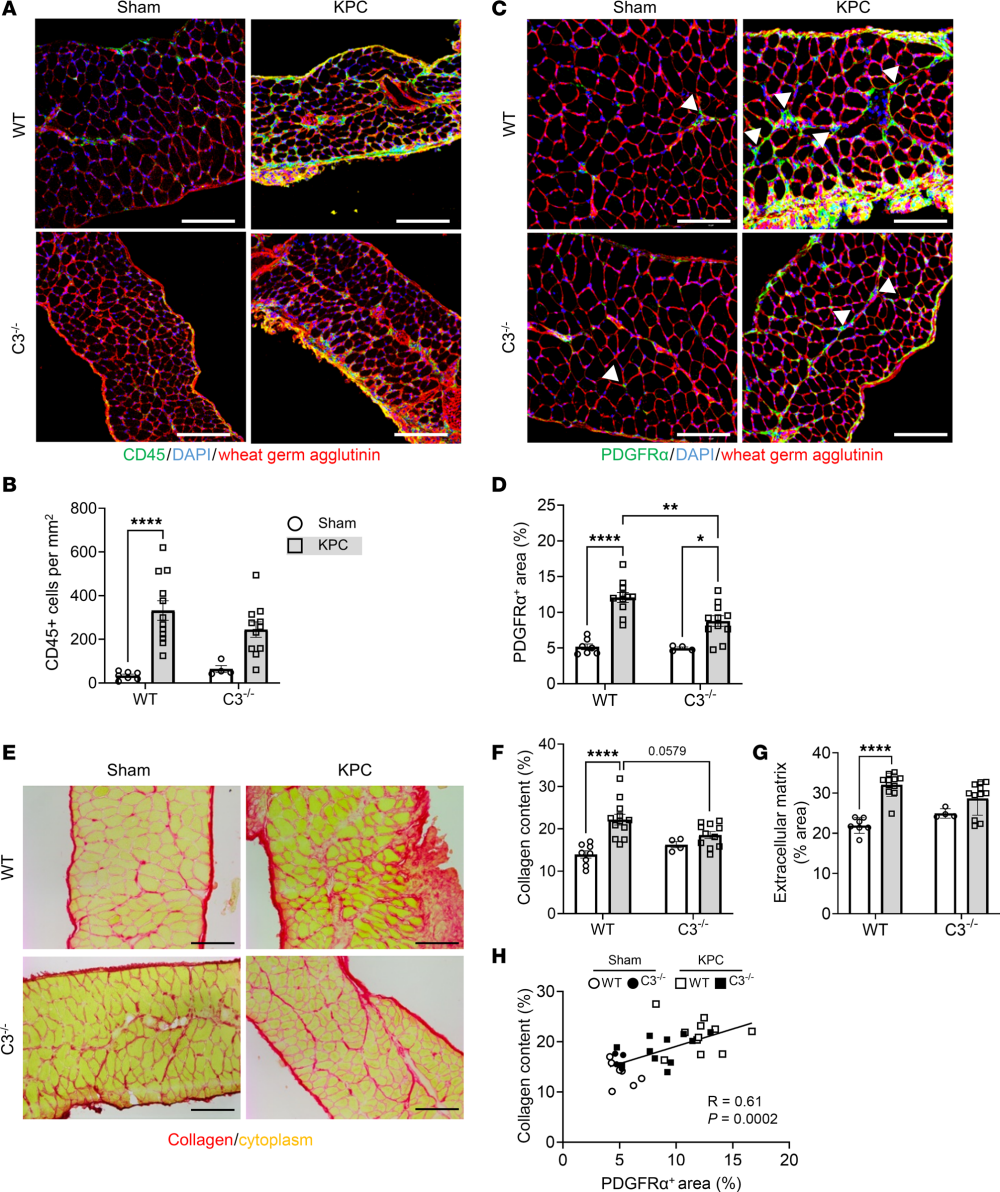
Deletion of C3 attenuates cell infiltration in diaphragm of KPC tumor–bearing mice. (**A**) Representative images of costal diaphragm cross sections stained for CD45, a pan-leukocyte marker (green); DAPI (blue); and wheat-germ agglutinin (red); scale bars: 100 μm. (**B**) Quantification of the density of infiltrating intramuscular leukocytes. (**C**) Representative images of costal diaphragm cross sections stained for platelet-derived growth factor receptor α (PDGFRα; green) as a marker of fibroadipogenic progenitor (FAP) cells (white arrowheads), DAPI (blue), and wheat germ agglutinin (red); scale bars: 100 μm. (**D**) FAP abundance was quantified as muscle area positive for PDGFRα. (**E** and **F**) Representative images of costal diaphragm cross sections subjected to Picrosirius red staining (scale bars: 100 μm) (**E**) for the quantification of total collagen content (**F**). (**G**) Muscle area occupied by extracellular matrix was quantified based on area positively stained for wheat germ agglutinin. (**H**) Correlation of collagen content and PDGFRα^+^ area. Data are presented as mean ± SEM, with individual data superimposed. Data are representative of *n* = 4–12 mice per group. Differences were assessed using a 2-way ANOVA with Šidák’s post hoc analysis (**B**, **D**, **F**, and **G**) and linear regression analysis (**H**). *P < 0.05, **P < 0.01, ****P < 0.0001.

We recently identified an increase in infiltrating leukocytes in the diaphragm of KPC mice that occurs prior to the onset of cachexia and continues throughout cachexia progression (32). Given that a key role of complement activation is to facilitate immune cell chemotaxis to local sites of complement activation (38), including in skeletal muscle (28, 39), we next stained diaphragm cross sections with CD45, a pan-leukocyte marker (Figure 8A), to determine whether the complement system mediates immune cell trafficking into the diaphragm of KPC mice. The diaphragm of WT KPC mice exhibited an approximately 10-fold increase in infiltrating leukocytes, which was attenuated in mice lacking C3 (Figure 8B). Since non-resolute inflammation can contribute to the expansion of fibroadipogenic progenitor (FAP) cells (40) and fibrotic remodeling of muscle, and we recently showed that leukocyte infiltration and inflammatory processes within the diaphragm of KPC mice precede the expansion of FAPs and fibrosis (32), we further assessed the impact of C3 deletion on the expansion of PDGFRα^+^ FAPs, ECM expansion, and collagen deposition within the diaphragm. While KPC tumors caused a robust increase in the abundance of PDGFRα^+^ FAP cells in the diaphragm of both genotypes, C3^–/–^ mice showed significantly attenuated (~43%) FAP cell abundance (Figure 8, C and D). Deletion of C3 also mitigated the expansion in muscle area occupied by collagen (Figure 8, E and F), and total ECM as measured via staining with wheat germ agglutinin (Figure 8G). Correlative analysis between FAP cells and muscle area occupied by collagen across genotypes revealed a significant, positive relationship (*P* = 0.0002, *R* = 0.61; Figure 8H), supporting the notion that FAP expansion is closely linked to the fibrofatty remodeling of respiratory muscles in pancreatic tumor–bearing hosts. Collectively, these findings suggest that in addition to complement regulating systemic wasting of muscles in response to PDAC, local complement activation in skeletal muscles proximal to the tumor, such as the diaphragm, may be a key mediator of leukocyte infiltration, FAP expansion, and replacement of muscle with fibrofatty tissue.

### Deletion of C3 preserves in vivo and ex vivo diaphragm function in response to KPC tumors.

**Figure 9:**
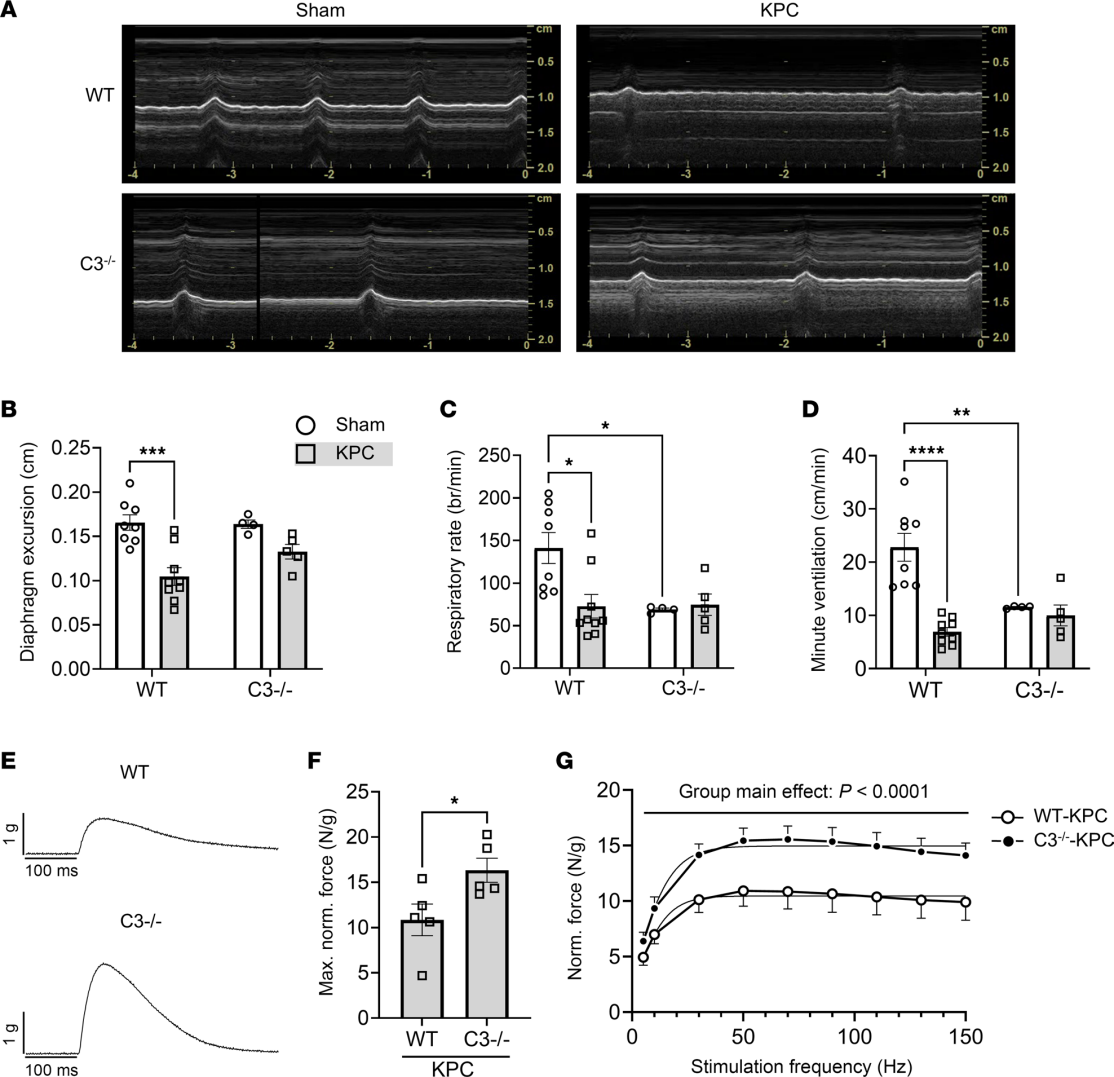
Deletion of C3 preserves in vivo and ex vivo diaphragm function during KPC tumor burden. (**A**) Representative M-mode ultrasonography traces of in vivo diaphragm contractions at day 14 after surgery in sham and KPC tumor–bearing mice. (**B**–**D**) M-mode ultrasonography was used to quantify diaphragm excursion (**B**), respiratory rate (**C**), and minute ventilation (**D**). (**E**) Representative tetanic force traces recorded from costal diaphragm of WT and C3^–/–^ KPC tumor–bearing mice. (**F**) Maximal normalized tetanic force recorded from costal diaphragm of WT and C3^–/–^ KPC tumor–bearing mice. (**G**) Force-frequency analysis of costal diaphragm demonstrated lower normalized force production in WT mice across a range of stimulation frequencies. Data are presented as mean ± SEM, with individual data superimposed. Data are representative of *n* = 4–9 mice per group. Differences were assessed using a 2-way ANOVA with Šidák’s post hoc analysis (**B**–**D** and **G**) and Student’s 2-tailed *t* test (**F**). *P < 0.05, **P < 0.01, ***P < 0.001, ****P < 0.0001.

Since C3 deletion mitigated several indices of diaphragm pathology in KPC mice, we subsequently determined whether this translates to preservation of respiratory and contractile function (12, 31, 32). In vivo diaphragm function was indirectly assessed, noninvasively, at study endpoint using M-mode ultrasonography (Figure 9A). KPC tumors reduced diaphragm excursion by about 37% in WT mice, but not in C3^–/–^ mice (Figure 9B). Respiratory rate (Figure 9C) and estimated minute ventilation (Figure 9D) were also decreased in WT mice but spared in C3^–/–^ mice. It is worth noting, however, that C3^–/–^ sham mice showed approximately 50% reduction in both respiratory rate and minute ventilation compared with WT sham mice, despite showing no overt phenotype and, to the best of our knowledge, exhibiting no known respiratory deficits. To evaluate diaphragm contractile function more directly, we measured the ex vivo contractile mechanics of costal diaphragm from WT and C3^–/–^ KPC mice (Figure 9, E–G). Relative to WT KPC mice, the maximal normalized force of the diaphragm from C3^–/–^ KPC mice was about 51% greater (Figure 9F). In addition, diaphragm from C3^–/–^ KPC mice generated greater tetanic forces across a range of stimulation frequencies compared with diaphragm from WT KPC mice (Figure 9G). Together with our histological data, these findings suggest that excessive complement activation in the diaphragm may be a key mediator of local inflammation and pathological replacement of muscle with fibrofatty tissue in response to PDAC that may contribute to muscle dysfunction — with strong translational relevance to patients.

## Discussion

The present study builds on previously published findings from our laboratory using muscle biopsies from cachectic PDAC patients, which identified a pathological progression toward the replacement of muscle with fat and fibrotic tissue that was associated with areas of immune cell infiltration and expansion of FAPs (16). Through unbiased proteomics, herein we further identified multiple proteins and biological processes associated with these pathologies, providing additional insight into underlying mechanisms that may be involved.

Using Masson’s trichrome staining on skeletal muscle cross sections, we previously discovered that the total muscle area occupied by collagen was increased in cachectic PDAC patients compared with non-cachectic PDAC patients and non-cancer controls, and was associated with poor survival (16). In the current study, using unbiased proteomics and follow-up IHC analyses, we found that the expansion of muscle area occupied by collagen in cachectic PDAC may be attributed to increased abundance of collagen IV. Collagen IV is highly abundant in skeletal muscle tissue and the primary collagen of the basement membrane, which surrounds muscle fibers and is critical for both structure and the physiological function of muscle (41). It is also the membrane under which satellite cells reside and plays a key role in the regeneration of muscle (42). Thus, any change in collagen IV levels has the potential to alter muscle structure and function, as well as satellite cell biology and muscle regeneration, all of which have been shown to be altered in the muscles of tumor-bearing hosts (43, 44). In contrast to collagen IV, proteomics analyses revealed decreased abundance of collagen type I α1 and α2 chain proteins, despite no changes in the total muscle area occupied by collagen type I via IHC. However, muscle from PDAC patients exhibited increased colocalization of collagen type I with the collagen hybridizing peptide (CHP), which has a high propensity for binding to unfolded collagen chains, and is used experimentally to detect, localize, and compare molecular damage and/or denaturation of collagen (20, 45). The finding that collagen I within muscle tissue is molecularly damaged in cachectic PDAC patients is not entirely surprising given that inflammatory processes were highly upregulated in muscles from these patients. Indeed, an abundance of literature exists supporting inflammation as a key trigger of collagen degradation within the ECM (46–48), with subsequent remodeling and expansion of the ECM occurring as an adaptive response to stabilize the damaged tissue. Consequently, muscle tissue inflammation and damage to collagen I, which confers tensile strength and rigidity to the muscle (49), have the potential to negatively impact muscle structure and function, including by increasing the susceptibility to contraction-induced injury that we have previously demonstrated occurs in respiratory muscles of tumor-bearing mice (12).

In addition to ECM remodeling, cachectic PDAC patients also displayed myofiber atrophy, which is a defining characteristic of cancer cachexia. Here, we found a significant and comparable, 33%–35%, decrease in the cross-sectional area of type I, type IIa, and type I/IIa hybrid fibers of cachectic PDAC patients compared with controls. This is in close agreement with previously published data in the rectus abdominis muscle from upper gastrointestinal or pancreatic cancer patients, which showed an approximately 26% decrease in the cross-sectional area of both type I and type IIa myofibers (50). We similarly did not find any evidence of a fiber type–specific difference in capillary contacts, with reduced capillary contacts for both type I and IIa fibers in PDAC patients compared with non-cancer controls. As alluded to previously, since capillaries deliver oxygen, nutrients, and growth factors that are essential to muscle health, a decrease in vascular density could be a trigger that disrupts muscle homeostasis. Indeed, in aging a decrease in vascular density has been linked to anabolic resistance and a decrease in muscle mass and strength (51, 52), each of which has been demonstrated in cancer cachexia. Moreover, adequate skeletal muscle capillarization is also vital for satellite cell function and muscle regeneration (53, 54), a process that is impaired in cancer cachexia (44). Thus, a decrease in muscle capillary density could be causative in several pathological features of cancer cachexia, which clearly warrants further study.

Our data suggest that both innate and adaptive immune system processes, and the complement system, are activated in muscle of cachectic PDAC patients and may play a key role in driving wasting, inflammation, and pathological remodeling of skeletal muscle. Excessive activation of complement is associated with muscle wasting and pathological remodeling in a number of conditions, including ischemia/reperfusion injury (6) and autoimmune and inflammatory diseases (7), through promoting inflammation and host tissue damage. Inappropriate activation of complement has also been identified as a driver of macrophage infiltration into skeletal muscle, and muscle weakness, in amyotrophic lateral sclerosis (39, 55), and is implicated in the pathogenesis of dysferlinopathy (8) and a number of other muscle diseases, including myasthenia gravis (56, 57); polymyositis, dermatomyositis, and inclusion body myositis (9, 10); and X-linked vacuolated myopathy (58). Our findings herein add to this body of knowledge by demonstrating complement activation in skeletal muscle of cachectic PDAC patients. Through our preclinical modeling of PDAC cachexia using the orthotopic KPC model, we demonstrate that the complement system also mediates leukocyte infiltration and pathological remodeling of the diaphragm — a murine muscle that we have shown recapitulates key aspects of muscle pathology observed in cachectic PDAC patients (33). Indeed, blockade of complement activation through deletion of C3 significantly deterred leukocyte infiltration into the diaphragm and inhibited the expansion of FAPs and the muscle ECM, supporting local complement activation as a key driver of respiratory muscle inflammation and pathological remodeling. While the initiating signals that incite complement activation in PDAC cachexia are not clear, local production/release of damage/danger-associated molecular patterns (DAMPs) from damaged or dying cells within the muscle could be involved (59). In this regard, we and others have previously demonstrated evidence of myofiber damage and sarcolemmal disruption in muscles of cachectic PDAC patients (16, 60). Our data also support an indirect role of complement activation in mediating the cachexia syndrome, as deletion of C3 also reduced whole-body wasting of muscle and fat, despite no effects on tumor size and minimal effects on the tumor transcriptome. However, since complement activation can be initiated by three pathways, and each converges on C3, our studies do not distinguish which of these pathway(s) are involved in cancer cachexia. Rather, the findings presented herein lay the foundation to further dissect the specific pathways involved in complement activation that mediate the cachexia phenotype.

The findings herein are supported by previous work that found that muscle, fat, and plasma complement levels associate with cachexia in pancreatic cancer patients. In this regard, increasing levels of complement component C4A mRNA in both muscle and adipose tissue were found to correlate with increased cancer weight loss grade in PDAC patients (61), while C3a, a cleavage product of C3, was recently shown to be significantly higher in plasma from cachectic pancreatic cancer patients with inflammation compared with those without cachexia (62). Notably, the complement pathway is also associated with cachexia induced by other cancer types and in response to chemotherapy. Indeed, in mice bearing colon adenocarcinoma 26 (C26) tumors, the complement pathway was identified as the most enriched pathway from genes upregulated in the muscles of both moderately and severely cachectic mice (63), with C3 protein levels found to also be increased in the muscle of cachectic C26 mice and in mice undergoing cachexia in response to FOLFIRI chemotherapy treatment (64). The complement system was also identified as an enriched pathway from genes upregulated in the muscles of cachectic mice bearing Lewis lung tumors (65). Thus, upregulation of the complement system is a common finding in cachectic tumor-bearing hosts.

Importantly, several FDA-approved complement inhibitors are currently available that are mostly used in rare diseases such as paroxysmal nocturnal hemoglobinuria, geographic atrophy, anti-neutrophil cytoplasmic autoantibody–associated vasculitis, and hereditary angioedema (66, 67). These inhibitors block complement activity at different stages of the activation pathway and include pegcetacoplan and eculizumab, which systemically bind and block cleavage of complement C3 and C5, respectively; Berinert and sutimlimab, which block early complement activation by inhibiting C1s/1r and mannose binding–associated serum proteases (MASPs); and avacopan, which inhibits the activation of C5a by antagonism of the C5a receptor. Interestingly, a randomized phase II trial at Roswell Park Comprehensive Cancer Center is currently testing the effect of pegcetacoplan when given in combination with the programmed death-1 (PD-1) inhibitor pembrolizumab, or when given in combination with pembrolizumab plus bevacizumab, an inhibitor of vascular endothelial growth factor (VEGF), in patients with ovarian, fallopian tube, or primary peritoneal cancer. The premise is based on the potential for pegcetacoplan to enhance antitumor activity by reducing immunosuppression in the tumor microenvironment when used in combination with immunotherapies. Thus, complement therapeutics hold potential as part of a multifaceted approach to cancer treatment.

In summary, we identified local complement deposition and activation in skeletal muscle of cachectic PDAC patients as a potentially key initiator of muscle inflammation and pathological replacement of muscle with fibrofatty tissue. Importantly, analyses were performed on skeletal muscle biopsies obtained from treatment-naive patients at the beginning of their tumor resection surgery, which supports the cancer and not the surgery or therapy as an explanation for these findings. Our findings are supported by the use of a complementary murine PDAC model, in which we demonstrate that respiratory muscles from PDAC mice deficient in complement activation show marked reductions in immune cell infiltration, expansion of fibroadipogenic progenitors, and ECM/collagen expansion in comparison with WT mice. Lastly, our data suggest a key role for complement activation in mediating whole-body cachexia, based on our finding that PDAC mice deficient in complement also showed attenuation in whole-body wasting of muscle and fat.

## Methods

### Sex as a biological variable.

Our tissue bank contained more muscle tissues from female patients naive to neoadjuvant therapy than it did from male patients naive to neoadjuvant therapy. To reduce the number of variables (such as sex and therapy) for the proteome study, we therefore chose to examine muscles collected from female patients only. Interestingly, although the lytic pathway is equally robust in female and male humans, the lytic pathway is not robust in female mice. Therefore, in our mouse studies, we selected male mice to study.

### Patient samples.

Skeletal muscle biopsies of the rectus abdominis were obtained from eligible patients following a prospective collection model, as previously described (16). Briefly, portions of each skeletal muscle sample were either flash-frozen in liquid nitrogen (for proteomic analyses) or embedded in optimal cutting temperature (OCT) compound and frozen in liquid nitrogen–cooled isopentane. All samples were stored at –80°C until further analyses.

The eligible population consisted of confirmed PDAC patients undergoing surgical resection with curative intent and weight-stable patients undergoing benign abdominal surgery as non-cancer controls at the University of Florida Pancreatic Surgical Center between March 2015 and September 2017. Demographics and clinicopathological details of patients are included in Table 1 and Supplemental Table 1. All patients were female, and all PDAC patients were naive to neoadjuvant therapy at the time of surgery and were defined as cachectic, with greater than 5% involuntary body mass loss and skeletal muscle depletion measured via CT-based skeletal muscle index (12). Samples from 8 PDAC patients and 6 non-cancer control patients were included for proteomic analyses. In cases in which there was insufficient sample remaining from a patient for follow-up histological analyses after the proteomics, additional samples from non-cancer control (*n* = 3) and PDAC (*n* = 2) patients, which were not included in the initial proteomic analysis, were added.

### Proteomic analysis.

The methodological workflow of proteomic analyses is displayed in Figure 1. Protein isolation and quantitative proteomics were performed by Cell Signaling Technologies, as previously described (68), and detailed methods are included in Supplemental Methods. Samples from cachectic PDAC patients and non-cancer controls were labeled with Tandem Mass Tag (TMT) reagents (Thermo Fisher Scientific), and labeled samples were fractionated using basic reverse-phase (bRP) fractionation chromatography. A total of 96 bRP fractions were collected over the entire gradient and multiplexed using a TMT10plex (Thermo Fisher Scientific). Samples were analyzed on an Orbitrap Fusion Lumos Tribrid mass spectrometer (Thermo Fisher Scientific). Peptide-spectral matching was performed using a target-decoy strategy and linear discriminant analysis at a 2% false discovery rate (FDR), with protein identification filtered to a 1% FDR. The mass spectrometry data were deposited in the ProteomeXchange database via the PRIDE repository (PXD047838).

Differences in protein abundance between PDAC and CTRL were determined by comparison of the summed signal-to-noise ratios, and significance was assessed using a nonparametric independent-samples 2-tailed *t* test. The *P* values were corrected with the Benjamini-Hochberg method, and relative protein abundance was expressed as fold change (FC) relative to CTRL. Proteins were classified as differentially expressed between PDAC and CTRL if they met the following criteria: detected peptides ≥2, adjusted *P* value (FDR) ≤ 0.05, median FC of >1.25 or < –1.25, same directional FC in ≥5 PDAC patients. The latter criterion for differential expression classification was implemented in an attempt to identify biological targets altered in a broader patient population.

### Animals.

Mice were given ad libitum access to standard chow and water and housed in a temperature- and humidity-controlled facility on a 12-hour light/12-hour dark cycle. Twelve-week-old male C57BL/6J mice (WT; stock 000664) and complement C3–null mice (C3^–/–^; stock 029661; ref. 69) were purchased from The Jackson Laboratory.

### Cancer cachexia model.

KPC FC1245 pancreatic cancer cells (obtained from David Tuveson, Cold Spring Harbor Laboratory, and isolated from the tumor of an *LSL-Kras^G12D/+^ LSL-Trp53^R172H/+^ Pdx-1-Cre* mouse) were maintained in DMEM supplemented with 10% FBS, 1% penicillin, and 1% streptomycin in a humidified chamber at 37°C and 5% CO_2_. For injection into mice, the pancreas was surgically exposed and the tail injected with 0.25 × 10^6^ KPC cells diluted in 50 μL of sterile PBS (KPC) or 50 μL of sterile PBS alone (sham). Mice were monitored daily and euthanized when WT KPC-bearing mice reached IACUC-mandated experimental endpoint, based on body condition score and tumor size.

### In vivo assessment of diaphragm function.

A wide-band phased-array ultrasound transducer (6S-RS, GE Healthcare) was positioned transverse on the abdomen of anesthetized mice, immediately distal to the xiphoid process, angled superiorly toward the diaphragm and thoracic cavity, ensuring no compression of the abdominal cavity. After a standardized duration of anesthesia was achieved, at least three M-mode video traces of the diaphragm were acquired (LOGIQ e Vet NextGen, GE Healthcare). Each video recorded 10 seconds of tidal breathing to measure excursion amplitude and breathing frequency, with measurements averaged per animal.

### Ex vivo muscle function assessment.

Ex vivo contractile function was assessed at the Physiological Assessment Core of the University of Florida as previously described (70) in WT and C3^–/–^ tumor-bearing mice. Briefly, at IACUC-mandated tumor endpoint, freshly isolated costal diaphragm strips were mounted on a force transducer (dual-mode lever system, Aurora Scientific) placed in a 22°C Ringer’s solution bath equilibrated with 95% O_2_ and 5% CO_2_. After optimal muscle length was determined, maximum isometric twitch and tetanic forces were measured using a single supramaximal stimulation and a 500-millisecond train at 150 Hz, respectively, with a 5-minute rest period between each set. Subsequently, diaphragm strips were stimulated at frequencies ranging from 5 to 150 Hz to establish a force-frequency relationship.

### Immunohistochemical analyses.

Transverse 7-μm-thick cross sections were cut from skeletal muscle samples previously embedded in OCT medium and frozen in liquid nitrogen–cooled isopentane. Sections were cut at –20°C and mounted onto uncoated glass slides. Slides were air-dried and stored at –80°C until further analysis.

Skeletal muscle cross sections from PDAC and CTRL patients were processed for gross morphological features including fiber type–specific myofiber size and typology, as well as fiber type–specific capillary content. Subsequent immunofluorescent assays were guided by bioinformatics of the unbiased proteomic analysis. Briefly, the abundance of collagen type I, collagen type IV, and collagen hybridizing peptide (CHP) was assessed as previously described (71). Expression of major histocompatibility complex class I (MHC-I) was quantified, and subsequently the abundance of CD3^+^ T cells and CD3^+^CD8^+^ cytotoxic T cells was assessed. Activation of the complement system was measured by quantification of the central regulator of complement, component C3, as well as formation of the terminal arm of complement, the membrane attack complex (MAC; C5b-9).

Cross sections of mouse limb (TA) and respiratory diaphragm muscle were subjected to hematoxylin and eosin (H&E) staining to assess gross morphology (33) and Picrosirius red staining to assess collagen content. Myofiber size was quantified in both muscles, whereas fiber typology was quantified in the diaphragm given its relatively heterogeneous fiber type profile compared with the TA. Infiltrating leukocytes were quantified through CD45 staining in both muscles, and the expansion of the fibroadipogenic progenitor cell population was assessed by staining for PDGFRα, as previously described (32). Detailed information on the methodologies and reagents used can be found in Supplemental Methods and Supplemental Table 2.

### Image acquisition and analysis.

Immunofluorescent analyses of PDAC and CTRL skeletal muscle samples, including fiber type–specific myofiber size and capillarization, expression of MHC-I, and deposition of complement C3 and C5b-9 (MAC), were acquired at ×100 total magnification with an upright fluorescent microscope equipped with a digital camera (DM5000B, Leica). Whole cross-sectional images of PDAC and CTRL samples stained for collagen I, collagen IV, CHP, and CD3^+^ and CD8^+^ T cells were acquired using a TCS SP8 confocal microscope (Leica). For colocalization analyses, images of collagen I, collagen IV, and CHP were acquired at ×200 total magnification (0.75 numerical aperture objective). Pixel intensity thresholds were set using single-labeled control samples as described previously (72), and all imaging parameters were kept constant for all samples. H&E and Picrosirius red staining was imaged with bright-field microscopy (DM5000B, Leica) at ×200 total magnification. All subsequent immunofluorescent staining of mouse TA and diaphragm was acquired as whole cross-sectional images on a confocal microscope (Leica) at ×200 total magnification. Colocalization analyses were performed on deconvolved images using LAS X software (Leica). All other image analysis was performed in Fiji (https://fiji.sc).

### RNA isolation, cDNA synthesis, quantitative reverse transcriptase PCR, and RNA sequencing.

For RNA isolation, TA muscles or KPC tumors were homogenized in Trizol, followed by chloroform extraction, isopropanol precipitation, and DNase treatment (AM1906, Invitrogen). Integrity of RNA was assessed with a Bioanalyzer 2100 (Agilent Technologies). From TA muscles, 1 μg of RNA was reverse-transcribed using the iScript Advance cDNA Synthesis kit (Bio-Rad). Cycle thresholds were measured via fluorometric PCR (QuantStudio 3, Applied Biosystems) on the generated cDNA using TaqMan probes listed in Supplemental Table 2 and quantified using the 2^–ΔΔCt^ method with *18S* as the reference gene. RNA from KPC tumors was sent to Novogene (Sacramento, California, USA) for RNA-Seq analysis. A total of 1 μg of RNA per sample was used to generate sequencing libraries, which were sequenced on Novogene’s Illumina NovaSeq 6000 (2 × 150 bp) to achieve at least 40 million reads per sample. Paired-end reads were aligned to the *Mus musculus* genome (mm39) using STAR (v2.5.; https://github.com/alexdobin/STAR) and annotated with HTSeq counts (v0.6.1.; https://github.com/htseq/htseq). Differential expression was analyzed with DESeq2 (v1.20.0.; https://bioconductor.org/packages/DESeq2/), and adjusted *P* values were calculated using the Benjamini-Hochberg method. Genes with adjusted *P* value less than 0.05 were considered differentially expressed. RNA-Seq data from KPC tumors are available at GEO GSE274179.

### Extraction of complement genes from diaphragm RNA-Seq data.

Genes involved in complement signaling were extracted from our recently published bulk RNA-Seq dataset from diaphragm muscles of sham mice and from KPC tumor–bearing mice collected at various time points throughout the cachexia trajectory (ref. 32 and GSE271521).

Bioinformatic enrichment analyses were performed using DAVID (73), STRING (74), and Ingenuity Pathway Analysis (75) platforms.

### Statistics.

Statistical analyses were performed using GraphPad Prism (v8). Data were tested for normality with the Shapiro-Wilk test. Comparisons between 2 groups were made with a Student’s 2-tailed *t* test or Mann-Whitney *U* test. For comparisons involving more than 2 groups, a 2-way ANOVA was performed with Šidák’s post hoc analysis. Correlations were assessed using linear regression. *P* values less than 0.05 were considered significant.

### Study approval.

The inclusion of human subjects was approved by the University of Florida Institutional Review Board, and written informed consent was obtained from all participants. All animal procedures were performed in accordance with NIH guidelines and with the approval of the University of Florida IACUC.

### Data availability.

All data generated in this study are available in public databases (RNA-Seq: Gene Expression Omnibus [GEO] GSE274179; mass spectrometry: ProteomeXchange database PXD047838) or in Supplemental Data File 3 as supporting data values.

## Author contributions

ACD, SMJ, and ARJ conceived the study. ACD, JBD, CSC, CA, SMJ, and ARJ participated in data acquisition, analysis, and interpretation. JGT collected the human samples. ACD, JBD, CA, JGT, SMJ, and ARJ drafted the manuscript and have primary responsibility for final content. All authors read and approved the submitted version and have agreed to be personally accountable for their own contributions and to ensure that questions related to the accuracy or integrity of any part of the work are appropriately investigated and resolved.

## Supplementary Material

Supplemental data

Supporting data values

## Figures and Tables

**Table 1:**
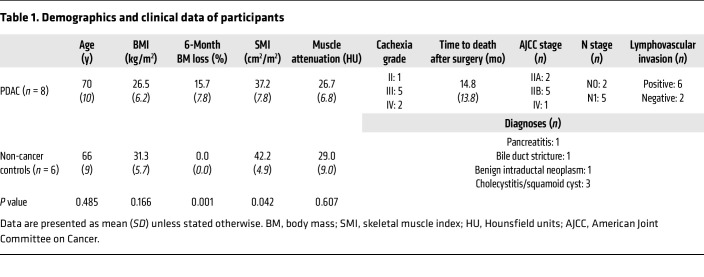
Demographics and clinical data of participants

## Version 1 04/08/2025

In-Press Preview

## Version 2 06/16/2025

Electronic publication
