# Supplementary material for: Complement pathway activation mediates pancreatic cancer–induced muscle wasting and pathological remodeling
Source: J Clin Invest. 2025 Apr 8;135(12):e178806. doi: 10.1172/JCI178806 (PMC12165795; doi:10.1172/JCI178806)
Supplement: Supplemental data [file jci-135-178806-s287.pdf]

Supplemental Table S1. Expanded human subject demographics and clinical data

	Age (y)	BMI (kg/m^2)	6-month BM Loss (%)	SMI (cm^2/m^2)	Muscle Attenuation (HU)	Cachexia Grade	Time to Death Post Surgery (m)	AJCC Stage (n)	N Stage (n)	Lymphovascular Invasion (n)
PDAC (n=10)	68 (*10*)	27.1 (*6.4*)	14.6 (*8.1*)	38.0 (*7.1*)	26.3 (*6.8*)	I: 1<br>II: 1<br>III: 5<br>IV: 3	13.0 (*12.8*)	IIA: 2<br>IIB: 7<br>IV: 1	N0: 2<br>N1: 7	Positive: 8<br>Negative: 2
						**Diagnoses (n)**				
Non-Cancer Controls (n=9)	59 (*14*)	29.4 (*5.6*)	1.6 (*3.7*)	42.6 (*4.1*)	33.3 (*9.7*)	Pancreatitis: 2<br>Bile Duct Stricture: 1<br>Benign Intraductal Neoplasm: 1<br>Cholecystitis/Squamoid Cyst: 5				
P-value	*0.115*	*0.433*	*0.001*	*0.035*	*0.133*					

Data are presented as mean (*SD*) unless stated otherwise.

Supplemental Table S2. Immunohistochemical antibodies, reagents, and oligonucleotides

	Primary			Secondary			Tertiary		
**Target**	**Antibody**	**Conc.**	**Cat. #; Vendor**	**Reagent**	**Conc.**	**Cat.#; Vendor**	**Reagent**	**Conc.**	**Cat.#; Vendor**
**CD3**	Mouse anti-human CD3 IgG_1	1:25	M7245; DAKO	Goat anti-mouse IgG, Biotin-SP	1:500	#115065166; Jackson Immunoresearch	Streptavidin, Alexa Fluor 594	1:75	W11262; Invitrogen
**CD45**	Rat anti-mouse CD45 IgG_{2b}	1:200	553076; BD Pharmingen	Goat anti-rat IgG, Alexa Fluor 488	1:300	A11006; Invitrogen			
**CD8**	Rabbit anti-human CD8 IgG	1:33	Ab4055; Abcam	Goat anti-rabbit IgG, Alexa Fluor 488	1:300	A11008; Invitrogen			
**Collagen (degraded/ remodeled)**	Collagen hybridizing peptide, 5-fam conjugate	20μM	F-CHP; 3Helix						
**Collagen Type I**	Mouse anti-collagen I IgG_1	1:25	8-3A5; Development al Studies Hybridoma Bank	Goat anti-mouse IgG_1, Alexa Fluor 555	1:300	A21127; Invitrogen			
**Collagen Type IV**	Mouse anti-collagen IV IgG_1	1:50	M3F7; Development al Studies Hybridoma Bank	Goat anti-mouse IgG_1, Alexa Fluor 555	1:300	A21127; Invitrogen			
**Complement C3**	Rabbit anti-human complement C3, Alexa Fluor 488 conjugate	1:33	Ab196458; Abcam						
**Complement C3**	Rabbit anti-complement C3, FITC conjugate	1:50	Ab4212; Abcam						
**DNA**	4',6-Diamidino-2-Phenylindole, Dihydrochloride	1:10,0 00	D1306; Invitrogen						

Supplemental Table S2. Immunohistochemical antibodies, reagents, and oligonucleotides (continued)									
	**Primary**			**Secondary**			**Tertiary**		
**Target**	**Antibody**	**Conc.**	**Cat. #; Vendor**	**Reagent**	**Conc.**	**Cat.#; Vendor**	**Reagent**	**Conc.**	**Cat.#; Vendor**
**Endothelial cells (human)**	ULEX Europeaus Agglutinin I, Rhodamine conjugate	1:200	RL1062; Vector Labs						
**Extracellular Matrix**	Wheat germ agglutinin-Alexa Fluor 594 conjugate	1:300	W11262; Invitrogen						
**Laminin**	Rabbit anti-laminin IgG	1:50	L9393; Sigma Aldrich	Goat anti-rabbit IgG, Alexa Fluor 555	1:300	A-21428; Invitrogen			
**Major Histocompatability Complex Class I**	Mouse anti-major histocompatability complex class I IgG_{2a}	1:100	MA5-11723; Thermo Fisher	Goat anti-mouse IgG, Alexa Fluor 488	1:300	A32723; Invitrogen			
**Membrane Attack Complex (C5b-9)**	Rabbit anti-C5b-9 IgG	1:100	Ab55811; Abcam	Goat anti-rabbit IgG Poly-HRP	Neat	B40943; Invitrogen	Alexa Fluor 488 Tyramide	1:100	B40943; Invitrogen
**Myosin Heavy Chain I**	Mouse anti-myosin heavy chain I IgG_{2b}	1:25	BA-D5; Developmental Studies Hybridoma Bank	Goat anti-mouse IgG_{2b}, Alexa Fluor 488	1:300	A21141; Invitrogen			
**Myosin Heavy Chain IIa**	Mouse anti-myosin heavy chain IIa IgG_1	1:25	SC-71; Developmental Studies Hybridoma Bank	Goat anti-mouse IgG_1, Alexa Fluor 350	1:300	A21120; Invitrogen			
**Platelet Derived Growth Factor Receptor Alpha**	Goat anti-mouse PDGFRα IgG	1:200	AF1062; R&D Systems	Donkey anti-goat IgG, Alexa Fluor 488	1:1000	A32814; Invitrogen			

Supplemental Table S2. Immunohistochemical antibodies, reagents, and oligonucleotides (continued)		
**Gene**	**Assay ID**	**Cat. #; Vendor**
**Fbxo31**	Mm00505343_m1	4331182; Thermo Fisher
**Fbxo32**	Mm00499523_m1	4331182; Thermo Fisher
**Trim63**	Mm01185221_m1	4331182; Thermo Fisher
**Psmd8**	Mm00466055_g1	4331182; Thermo Fisher
**Ubc**	Mm02525934_g1	4331182; Thermo Fisher
**Ctsl**	Mm00515597_m1	4331182; Thermo Fisher
**Gabarapl1**	Mm00457880_m1	4331182; Thermo Fisher
**Sqstm1**	Mm00448091_m1	4331182; Thermo Fisher
**18S**	Hs03003631_g1	4448484; Thermo Fisher

SUPPLEMENTAL METHODOLOGY

Proteomic analysis

Protein isolation and peptide generation

Proteins were extracted from skeletal muscle tissue in lysis buffer (9M urea, 20mM Hepes pH 8.5, 10mM sodium pyrophosphate, 1mM sodium orthovanadate, 1mM beta-glycerophosphate) using a handheld homogenizer. Samples were then sonicated for 30 sec, then centrifuged at 12000xG for 15 min. Protein concentrations were measured using the BCA Protein Assay Kit (Pierce). Protein samples (100ug) were reduced in 45mM DTT (1:10, volume/sample volume) and incubated for 30 min at 55°C. Lysates were allowed to cool and were then alkylated with 100mM iodiacetamide (1:10, volume/sample volume) at room temperature (RT), in the dark. Samples were then diluted in 20mM HEPES buffer pH 8 (1:4, volume /sample volume). Protein samples were digested with LysC (1:250, protease/protein mass) for 4 hours at 37°C, then trypsin (1:40, protease/protein mass) overnight at RT. Following digestion, samples were purified over Sep-Pak C18 columns (Waters, Milford, MA) and the eluted peptides were lyophilized.

Tandem Mass Tag labeling and basic pH reversed-phase fractionation

Tandem mass tag (TMT) isobaric reagents were acquired from ThermoFisher Scientific (Waltham, MA). Peptides (100ug) were resuspended in 100ul of 200mM HEPES buffer pH 8.5, at which point 40ul was combined with one of ten distinct TMT labels, resuspended in 100% acetonitrile (ACN). Samples were incubated for 1 hour at RT then quenched with hydroxylamine for 15 min at RT. After quenching, 40ug of each of the ten samples were combined, dried, desalted over a StageTip, and analyzed for labeling and mixing. The TMT labels used were as follows: 126, PDAC; 127N, PDAC; 127C, PDAC; 128N, PDAC; 128C, PDAC; 129N, PDAC; 129C, PDAC; 130N, PDAC; 130C, pooled controls 1-3; and 131, pooled controls 4-6. Peptide labelling efficiency was 96.65%.

Samples were resuspended in basic pH reversed-phase (bRP) buffer A (10 mM NH_4HCO_2, pH10, 5% ACN) and separated on a Zorbax Extended C18 bRP column (2.1 × 150 mm, 5 μm, #773700-902, Agilent, Santa Clara, CA) using a gradient of 10–40% bRP buffer B (10 mM NH_4HCO_2, pH10, 90% ACN) over 50 min at a flow rate of 200 $\mu l \cdot min^{-1}$. A total of 96 fractions were collected before further concatenation into 12 final fractions. Each fraction was then dried and desalted over a C18 StageTip prior to analysis by mass spectrometry.

LC-MS/MS analysis

All samples were analyzed on an Orbitrap Fusion mass spectrometer (Thermo Fisher Scientific, San Jose, CA) coupled with a Proxeon EASY-nLC 1000 liquid chromatography (LC) pump (Thermo Fisher Scientific). Peptides were loaded directly onto a 50 cm x 100 µm PicoFrit capillary column packed with C18 reversed-phase resin. For each analysis, we loaded approximately 2.7 μg onto the column. Peptide were separated using a 150 min gradient of 6-30% acetonitrile in 0.125% formic acid with a flow rate of 150 nL·min. Each analysis used an MS3-based TMT method(9, 13), which has been shown to reduce ion interference compared to MS2 quantification (11). The scan sequence began with an MS1 spectrum [Orbitrap analysis, resolution 120000, 200-1500 Th, automatic gain control (AGC) target 1 x 106, maximum injection time 150ms]. The top 10 precursors were then selected for MS2/MS3 analysis. MS2 analysis consisted of collision-induced dissociation (CID), quadrupole ion trap analysis, automatic gain control (AGC) 2 x 103, normalized collision energy (NCE) 35, q-value 0.25, and maximum injection time 100 ms. Following the acquisition of each MS2 spectrum, an MS3 spectrum was collected as previously described (9). MS3 precursors were fragmented by high energy collisional dissociation (HCD) and analyzed using an Orbitrap Fusion MS (NCE 65, AGC 1.5 x 105, maximum injection time 250 ms, isolation specificity 0.80 Th, resolution 60000 at 400 Th).

Peptide and protein identification

Following data collection, MS/MS spectra were processed using SEQUEST and the core platform from Harvard University. Searches were performed against the most recent update of the Uniprot *Homo sapiens* database with mass accuracy of ± 50 ppm and 0.02 Da for product ions. Peptide-spectral matching was performed using a target decoy strategy and linear discriminant analysis (LDA) set to a false discovery rate (FDR) of 2%, while considering XCorr, deltaCn, Rsp, and discriminant score. Protein identification was filtered to a 1% FDR.

Histochemistry and immunohistochemical analyses

Detailed information on antibodies and reagents utilized in immunohistochemical analyses can be found in Supplementary Table S1.

Picrosirius Red

Slides were air dried for at least 30 min, then fixed in Bouin's fluid for 30 min at 37°C. Following a 3 min rinse in ddH_2O, slides were incubated in picrosirius red solution (0.1% Direct Red 80 in saturated picric acid) for 90 min at RT. Slides were then rinsed in 0.5% glacial acetic acid and picric alcohol for 30 sec each. Following dehydration through a graded ethanol series, slides were cleared with xylene and mounted with mounting media (Cytoseal XYL, Thermo Fisher).

Fiber type-specific myofiber size

Slides were thawed at RT for 30 min and rehydrated in 1X phosphate-buffered saline (PBS) for 3 min. After blocking for 1 h in 10% normal goat serum, slides were incubated for 90 min at RT in the following primary antibodies: laminin (#L9393, Sigma, St. Louis, MO), myosin heavy chain (MyHC) I [BA.D5, mouse monoclonal supernatant, Developmental Studies Hybridoma Bank (DSHB)], and MyHC IIa (SC.71, mouse monoclonal supernatant, DSHB). Following a series of washes in PBS, slides were incubated for 60 min at RT in the following

secondary antibodies: goat anti-mouse IgG_{2b} Alexa Fluor (AF) 488 (#A21141, Invitrogen, Carlsbad, CA), goat anti-mouse IgG_1 AF350 (#A21120, Invitrogen), and goat anti-rabbit IgG1 Rhodamine Red (#R6394, Invitrogen). Slides were washed in PBS and mounted with fluorescent mounting medium (Prolong Diamond, #P36965, Invitrogen).

Fiber type-specific capillarization

After fixation in ice-cold acetone for 10 min at -20°C slides were incubated in 3% H_2O_2 for 7 min at RT, then blocked in 10% normal goat serum for 60 min at RT. Slides were incubated overnight in a cold-room at 4°C in primary antibodies detected against MyHC I (BA.D5) and laminin (#L9393, Sigma) and rhodamine-conjugated ULEX Europeaeus Agglutinin I (#RL-1062, Vector Labs) to visualize endothelial cells. The next day, slides were incubated goat anti-mouse IgG_{2b} AF488 (#A21141, Invitrogen), goat anti-rabbit IgG AF647 (#A21244, Invitrogen) for 60min at RT. Following a series of washes in PBS, slides were costained with 4′,6-diamidino-2-phenylindole (DAPI) for 10min, then mounted with fluorescent mounting medium.

Collagen composition and remodeling

Slides were fixed in ice cold acetone for 10 min at -20°C and blocked in 2.5% normal horse serum for 60 min at RT. CHP 5-fam conjugate (3Helix, Salt Lake City, UT) was diluted to a working concentration of 20µM in PBS, denatured at 80°C for 5 min, then cooled on ice for 2 min prior to the addition of a primary antibody directed against collagen type I (8-3A5, mouse monoclonal supernatant, DSHB) or collagen type IV (M3F7, mouse monoclonal supernatant, DSHB) diluted in 2.5% normal horse serum. Slides were incubated in the CHP and primary antibody mixture overnight in a cold-room at 4°C. The next day slides were incubated for 90 min at RT in goat anti-mouse IgG_1 Rhodamine Red (#R6393, Invitrogen), washed in PBS, and mounted with fluorescent mounting medium.

Major histocompatibility complex class I

After fixation in ice cold acetone for 10 min at -20°C slides were incubated in 3% H_2O_2 for 7 min at RT, then blocked in 10% normal goat serum for 60 min at RT. Primary antibody directed against major histocompatibility complex class I (MHC-I, #MA5-11723, Clone: W6/32, ThermoFisher) was applied overnight at 4°C. The next day slides were washed in PBS, incubated in goat anti-mouse IgG AF488 (#A11001, Invitrogen) for 60 min at RT, and costained with DAPI prior to being mounted with fluorescent mounting medium.

Infiltrating T cells

Slides were stained for CD3, a marker of all mature T cells, and CD8, a marker of cytotoxic T cells. Following fixation in ice cold acetone for 10 min at -20°C, slides were incubated in 3% H_2O_2 for 7 min at RT, then blocked in 2.5% normal horse serum for 60 min at RT. Slides were incubated overnight in a cold room at 4°C in primary antibodies directed against CD3 (#M7254, Clone: F7.2.38, DAKO, Glostrup, Denmark) and CD8 (#ab4055, Abcam). The next day slides were incubated in goat anti-mouse IgG biotin-SP conjugate (#115-065-205, Jackson ImmunoResearch, West Grove, PA) and goat anti-rabbit AF488 (#11008, Invitrogen) for 60 min at RT, followed by an incubation in streptavidin-AF594 conjugate (#S32356, Invitrogen) and DAPI for 60 min at RT prior to being mounted with fluorescent mounting medium.

Activation of complement system

Slides were stained for complement component 3 (C3) and the membrane attack complex (MAC). To assess C3 deposition, slides were blocked for 30 min at RT in Pierce Super Block (#37580, ThermoFisher) then incubated for 2h at RT in AF488 conjugated anti-C3 primary antibody (#ab196458, Abcam, Cambridge, MA. Following a series of washes in PBS, slides were costained with 4′,6-diamidino-2-phenylindole (DAPI) then mounted with fluorescent mounting medium. To assess MAC deposition, slides were blocked in 10% normal goat serum for 60 min at RT, then incubated overnight in a cold-room at 4°C in primary antibody directed against C5b-9

(#ab55811, Abcam). The next day, slides were incubated for 60 min at RT in goat anti-rabbit IgG Poly-HRP included with the tyramide signal amplication (TSA) kit (#B40943, Invitrogen). Following a series of washes in PBS, slides were reacted with AF488 tyramide solution (included in TSA kit), costained with DAPI, and mounted with fluorescent mounting medium.

Infiltrating leukocytes

Slides were stained for the common leukocyte antigen, CD45. Sections were fixed in 4% PFA for 5 min at RT and permeabilized in 0.1% TX-100 for 10 min. Slides were incubated for 60 min at RT in blocking solution (3% bovine serum albumin, 10% NGS) then incubated in rat anti-mouse CD45 (553076, BD Bioscience) in blocking buffer overnight at 4°C. Slides then washing in PBS and incubated in goat anti-rat IgG AF488 (A11006, Invitrogen) for 60 min at RT. Sections were incubated in DAPI for 10 min and mounted in fluorescent mounting medium.

Fibro-adipogenic progenitor cells

Slides were stained for the established FAP marker, platelet-derived growth factor receptor α (PDGFRα) as previously described (7). Briefly, slides were fixed in 4% PFA for 10 min and blocked for 60 min in blocking solution (5% donkey serum, 0.3% TX-100). Slides were incubated in goat anti-mouse PDGFRα IgG (AF1062, R&D Systems) in blocking solution overnight at 4°C. The next day, slides were washed in PBS and incubated in donkey anti-goat IgG AF488 (A32814, Invitrogen) and AF594-conjugated wheat germ agglutinin (W11262, Invitrogen) for 2 h at RT. Slides were incubated in DAPI for 10 min and mounted with fluorescent mounting medium.

REFERENCES

1. **Anders S, Pyl PT, and Huber W**. HTSeq--a Python framework to work with high-throughput sequencing data. *Bioinformatics* 31: 166-169, 2015.
2. **Andrews S**. FastQC: A Quality Control Tool for High Throughput Sequence Data Babraham Bioinformatics. http://www.bioinformatics.babraham.ac.uk/projects/fastqc/. 2019].
3. **Benjamini Y, and Hochberg Y**. Controlling the false discoveryrate: a practical and powerful approach to multiple testing. *Journal ofRoyal Statistical Society: Series B* 57: 289-300, 1995.
4. **Dobin A, Davis CA, Schlesinger F, Drenkow J, Zaleski C, Jha S, Batut P, Chaisson M, and Gingeras TR**. STAR: ultrafast universal RNA-seq aligner. *Bioinformatics* 29: 15-21, 2013.
5. **Durinck S, Moreau Y, Kasprzyk A, Davis S, De Moor B, Brazma A, and Huber W**. BioMart and Bioconductor: a powerful link between biological databases and microarray data analysis. *Bioinformatics* 21: 3439-3440, 2005.
6. **Judge SM, Deyhle MR, Neyroud D, Nosacka RL, D'Lugos AC, Cameron ME, Vohra RS, Smuder AJ, Roberts BM, Callaway CS, Underwood PW, Chrzanowski SM, Batra A, Murphy ME, Heaven JD, Walter GA, Trevino JG, and Judge AR**. MEF2c-dependent downregulation of Myocilin mediates cancer-induced muscle wasting and associates with cachexia in cancer patients. *Cancer Res* 2020.
7. **Kopinke D, Roberson EC, and Reiter JF**. Ciliary Hedgehog Signaling Restricts Injury-Induced Adipogenesis. *Cell* 170: 340-351.e312, 2017.

8. **Love MI, Huber W, and Anders S**. Moderated estimation of fold change and dispersion for RNA-seq data with DESeq2. *Genome biology* 15: 550, 2014.

9. **McAlister GC, Nusinow DP, Jedrychowski MP, Wuhr M, Huttlin EL, Erickson BK, Rad R, Haas W, and Gygi SP**. MultiNotch MS3 enables accurate, sensitive, and multiplexed detection of differential expression across cancer cell line proteomes. *Anal Chem* 86: 7150-7158, 2014.

10. **Nosacka RL, Delitto AE, Delitto D, Patel R, Judge SM, Trevino JG, and Judge AR**. Distinct cachexia profiles in response to human pancreatic tumours in mouse limb and respiratory muscle. *J Cachexia Sarcopenia Muscle* 2020.

11. **Paulo JA, O'Connell JD, and Gygi SP**. A Triple Knockout (TKO) Proteomics Standard for Diagnosing Ion Interference in Isobaric Labeling Experiments. *J Am Soc Mass Spectrom* 27: 1620-1625, 2016.

12. **Piras IS, Krate J, Schrauwen I, Corneveaux JJ, Serrano GE, Sue L, Beach TG, and Huentelman MJ**. Whole transcriptome profiling of the human hippocampus suggests an involvement of the KIBRA rs17070145 polymorphism in differential activation of the MAPK signaling pathway. *Hippocampus* 27: 784-793, 2017.

13. **Ting L, Rad R, Gygi SP, and Haas W**. MS3 eliminates ratio distortion in isobaric multiplexed quantitative proteomics. *Nat Methods* 8: 937-940, 2011.
